# Comparative Plastomics of Plantains (*Plantago*, Plantaginaceae) as a Tool for the Development of Species-Specific DNA Barcodes

**DOI:** 10.3390/plants13192691

**Published:** 2024-09-25

**Authors:** Furrukh Mehmood, Mingai Li, Alessio Bertolli, Filippo Prosser, Claudio Varotto

**Affiliations:** 1 Ecogenomics Unit, Research and Innovation Centre, Fondazione Edmund Mach, 38098 San Michele all’Adige, Italy; 2 National Biodiversity Future Center (NBFC), 90133 Palermo, Italy; 3 Fondazione Museo Civico di Rovereto, 38068 Rovereto, Italy

**Keywords:** plantains, plastome, indels, medicinal plants, mutational hotspots, phylogeny, Plantaginaceae, substitutions, *Plantago*, molecular markers

## Abstract

*Plantago* (plantains, Plantaginaceae) is a cosmopolitan genus including over 250 species used as functional foods, forage, and traditional medicine. Among them, *Plantago lanceolata* is commonly used as an ingredient of herbal products, but the close similarity to other *Plantago* species can cause misidentifications with potentially serious consequences for product safety/quality. To test the possibility of developing species-specific barcoding markers, we de novo assembled plastome sequences of individuals of *Plantago argentea*, *Plantago atrata*, *P. lanceolata*, and *Plantago maritima*. These genomes were characterized in comparison with both previously sequenced conspecific accessions and other publicly available plastomes, thus providing an assessment of both intraspecific and interspecific genetic variation in *Plantago* plastomes. Additionally, molecular evolutionary analyses indicated that eleven protein-coding genes involved in different plastid functions in *Plantago* plastomes underwent positive selection, suggesting they might have contributed to enhancing species’ adaptation during the evolutionary history of *Plantago*. While the most variable mutational hotspots in *Plantago* plastomes were not suitable for the development of species-specific molecular markers, species-specific polymorphisms could discriminate *P. lanceolata* from its closest relatives. Taken together, these results highlight the potential of plastome sequencing for the development of molecular markers to improve the identification of species with relevance in herbal products.

## 1. Introduction

*Plantago* is a cosmopolitan genus that has diversified into over 250 species, which are usually anemophilous herbs or rarely subshrubs, perennial or annual, and concentrated in temperate and high-elevation tropical regions [1,2,3]. The taxonomy of *Plantago* is notoriously difficult, owing to the relatively few morphological characters available and its evolutionary trend toward morphological reduction and simplification [4,5]. Traditionally, several morphological features, including trichomes and seeds, as well as chemotaxonomic analyses [6,7,8], have been employed in attempts to identify and classify the species. However, none of these methods have yielded a conclusive result that is considered satisfactory, not even with 91 mainly morphological and embryological characters [2]. Furthermore, the existence of polyploidy [9,10], hybridization [10], and reticulate evolution [11] adds more difficulty to the understanding of evolutionary relationships in *Plantago*. Not surprisingly, therefore, early attempts aimed at determining the phylogenetic relationships among *Plantago* species with either morphological or molecular data from a limited number of loci provided strong support for the sister relationship between *Plantago* and *Littorella*, but the infrageneric relationships were still not fully resolved [2,11]. Only recently have phylogenomic studies adopting whole-chloroplast genome sequencing succeeded in the full resolution of the subgeneric structure, resulting in the recognition of four highly supported subgenera in the genus *Plantago*: *Bougueria* (Decne.) Rahn (previously regarded as a distinct monotypic genus; [2,12]), *Coronopus* (Lam. & DC.) Rahn, *Plantago* and *Psyllium* (Mill.) Harms & Reiche [4,13]. 

Some *Plantago* species hold significant value in the nutraceutical and pharmaceutical sectors because of the mucilaginous substance, psyllium, obtained from the seed husk. This product is recognized for its use as a functional food and dietary supplement, particularly for enhancing intestinal health [14,15]. In some regions, various *Plantago* species are used as ingredients in salads, soups, or baked products [16,17]. Additionally, certain species are utilized in animal feed to enhance health and reduce the reliance on antibiotics [18]. 

*P. lanceolata* is a common forb, native to Europe and Western Asia, with a cosmopolitan distribution and high adaptability [19,20,21,22]. The aerial parts of *P. lanceolata* exhibit properties that include wound-healing, anti-inflammatory, antibacterial, diuretic, and anti-asthmatic effects [23]. The consumption of juice extracted from *P. lanceolata*, when combined with wine or honey, is believed to provide relief from gout symptoms. Additionally, the crushed leaves of the plant, when mixed with salt, have been traditionally employed to treat arthritis symptoms [24]. At present, *P. lanceolata* is utilized in the treatment of mouth, throat, and upper respiratory tract conditions, as well as for topical application in skin diseases [25]. Many products, including teas and syrups, containing *P. lanceolata* have been introduced to the market, with a significant presence in Europe. The herb is recognized as safe for use in the pharmacopeias of several countries [26]. 

The market for herbal products has become increasingly prevalent around the world in recent decades, with the worldwide market for medicinal plants projected to reach a value of USD 5 trillion by 2050, with Europe being a significant driving force behind this growth [27,28]. Plantain (*Plantago* species) products, however, still show qualitative variability through the herbal medicinal value chain, affecting their impact and safety. A notable instance of plant misidentification, for instance, was documented in a study that detailed the cases of two patients who presented to hospital emergency rooms with symptoms such as palpitations, vomiting, nausea, and chest pressure, among other symptoms [29]. The investigation revealed that both these patients consumed raw material labeled as plantain (genus *Plantago*) that had been contaminated with *Digitalis lanata* due to misidentification in the field. The quality of manufactured herbal products is notoriously variable on a global scale, with significant inconsistencies in the quality of these products observed across different regions and suppliers. To address this issue, consistent and rigorous analytical approaches are necessary to accurately identify and monitor the quality of herbal products throughout the entire value chain [30]. The herbal medicine industry has considered DNA barcoding as a method that can be consistently applied in quality control over manufactured products and to identify medicinal materials to protect consumers from dishonest suppliers. In addition, this method can also be used to identify toxic herbal materials in life-threatening situations, prevent poisoning, and improve control procedures of herbal drug substances [3].

The structure and sequence of the chloroplast genome (or plastome) can be utilized to generate molecular markers that can be used in DNA barcoding [31]. Plastomes have been termed “super barcodes”, due to their comparatively conserved organization, gene content, adequate level of nucleotide substitution in protein-encoding genes, and uniparental inheritance, which make them excellent sources of genetic information for phylogenetic reconstruction and species identification at diverse taxonomic levels [32,33,34,35]. Currently, whole-chloroplast genomes are available from the National Centre for Biotechnology Information (NCBI) Organelle Genome Resources (accessed on 3 June 2024) and TreeBase (https://www.treebase.org/treebase-web/home.html; accessed on 3 June 2024) for approximately 110 of the about 250 *Plantago* species. While for about 21% of the species, multiple plastomes are available (for instance, in the case of *P. lanceolata*, for which five plastome sequences have been deposited), the intraspecific genetic variation for most species has not been determined. In the subgenus *Coronopus*, seven species have fully sequenced plastomes, including two accessions of *P. maritima* [4,13]. In the *Psyllium* subgenus, the plastomes of 11 species have been sequenced, among which two (*P. lanceolata* and *Plantago lagopus*) belong to section *Lanceifolia*, and one (*P. atrata*) from the sister section *Montana* [13]. Former phylogenies based on ITS and *trnL-F* sequence data retrieved with good statistical support the narrow endemic *Plantago leiopetela* from the islands of Madeira and Porto Santo as a sister to *P. lanceolata* [12,36]. In an unpublished study with higher taxonomic sampling, however, this relationship was only weakly supported [37]. Interestingly, in the latter study, a highly supported clade of European and Mediterranean species formed by *P. lanceolata*, *P. leiopetala*, *Pplantago loeflingii*, *P. argentea*, and *Plantago altissima* was recovered, although the topology among these species lacked significant support [37]. In the published version of the work, only *P. argentea* was included in the analyses and five subgenera were recognized [5].

Here, we aimed to explore in greater depth the intra- and interspecific diversity of the chloroplast genomes of some of the species most closely related to *P. lanceolata* and to apply this knowledge to develop highly robust and reliable barcoding markers for this important plantain species. With this goal in mind, we sequenced, assembled, and annotated the complete plastomes of single accessions of *P. argentea*, *P. atrata*, *P. lanceolata*, and *P. maritima* co-occurring in the same region of the Eastern Alps. By comparing the newly sequenced plastome accessions with those present in public databases, in addition to presenting a novel assessment of the evolutionary patterns shaping the selective landscape of *Plantago* plastomes and reassessing the *tempo* of the genus evolution, we addressed the placement of the novel *P. argentea* plastome in the *Plantago* phylogeny and particularly with respect to *P. lanceolata*.

## 2. Results

### 2.1. Organization and Characteristics of Plantago Plastid Genomes

**Figure 1:**
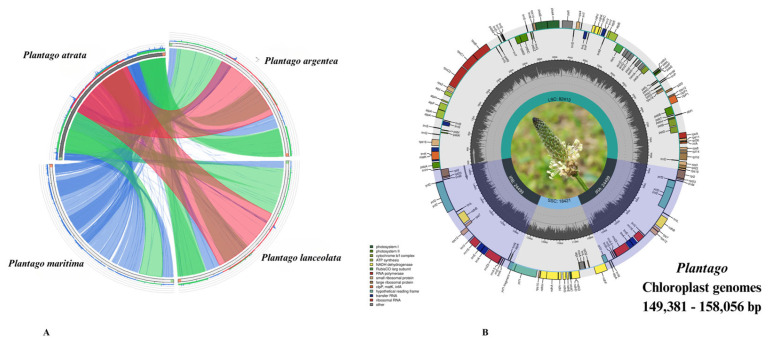
(**A**) Structural comparison of the four studied *Plantago* plastomes showing a high level of synteny and the lack of large rearrangements. The start and end points of the sequences are marked by green and orange blocks. The colored blocks outside the sequences refer to the score/max bit score ratio, with green ≤0.50, orange ≤0.75, and red >0.75. Blue blocks and chords represent the inverted repeats (IRs). (**B**) Consensus circular genome map of four studied *Plantago* plastomes. Genes drawn inside the circle are transcribed counterclockwise and those outside are clockwise. Different colors indicate the genes belonging to various functional groups. GC and AT content of the genome are plotted in light grey and dark, respectively, in the inner circle. Large single copy (LSC), inverted repeat A (IRa), and inverted repeat B (IRb) highlighted with color and small single copy (SSC) are shown in the circular diagram.

Our comparative analysis confirmed that *Plantago* species have similar plastid genome structures (Figure 1A and Table 1). 

All four *Plantago* plastomes exhibited a typical quadripartite structure containing a pair of inverted repeats (IR) regions (24,499–33,741 bp), an LSC region (81,909–82,680 bp) and an SSC region (8665–18,421 bp). The full-length variation between these genomes was about 8.6 kb (genome size: 149,381–158,056 bp). The assembled plastomes of *P. argentea*, *P. atrata*, *P. lanceolata*, and *P. maritima* had average coverage depths of 103×, 50×, 160×, and 80×, respectively. The total guanosine-cytosine (GC) content of the *de novo* assembled *Plantago* plastid genomes was 38.3%, as was that in the previously sequenced species. The GC content of the IR region was higher (43.6%) than the LSC (36.6%) and SSC (32.6%) regions, which could have been due to the occurrence of rRNA genes, which are known to contain GC-rich regions [38,39]. The plastomes of the *de novo* assembled *Plantago* accessions had 133–137 unique genes, whereas 18–22 genes were duplicated in the IR (Table 2 and Figure 1B). 

**Figure 2:**
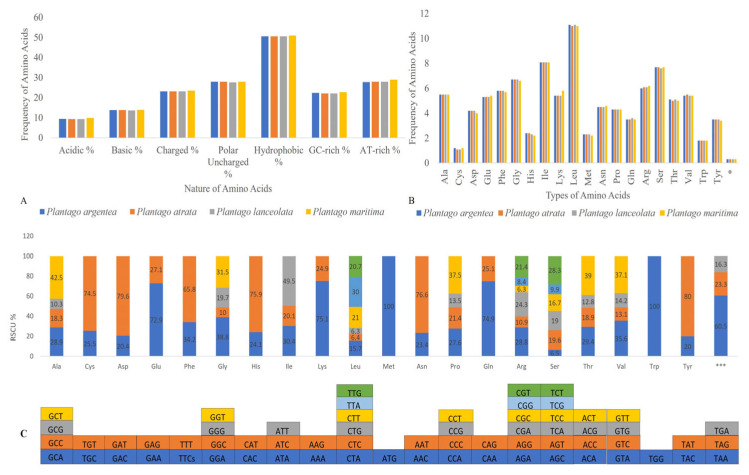
(**A**) Assessment of amino acid groups and (**B**) amino acid frequency comparison among *Plantago* species. (**C**) The codon content and RSCU value of 20 amino acids and stop codons in all protein-coding genes in the plastomes of *Plantago* species. The color of the histogram in (**C**) is consistent with the color of codons in the same panel. * and *** indicate the end of the protein and the stop codon, respectively.

Out of these 133–137 genes, 86–90 were protein-encoding, 38–39 were tRNA, and 8 rRNA genes were similarly present in all *Plantago* species. The IR regions contained 18–22 duplicated genes and out of these 7–11 were protein-encoding, 4 were rRNA, and 7 were tRNA genes. In all four studied *Plantago* species, the *ycf3* protein-coding gene contains two introns. Within the subgenus *Psyllium* (*P. argentea*, *P. atrata*, and *P. lanceolata*), the genes *rps16*, *atpF*, *rpoC1*, *clpP*, *petB*, *petD*, *rpl16*, *rpl2*, *ndhB*, *rps12*, and *ndhA* each contain one intron. However, in *P. maritima* (subgenus *Coronopus*), the only difference observed is the loss of introns in the *rpl2* and *clpP* genes as compared to the subgenus *Psyllium*. The *rps12* gene was trans-spliced with its 5′-end exons located in the LSC, while its two 3′-end exons were found in the IR. The tRNAs (52.8%) and rRNAs (55.2%) showed the highest GC content. Hydrophobic amino acids were abundant, while acidic amino acids were present in the smallest amount in the plastid genomes of all four studied *Plantago* species. These amino acids were coded by adenine-thymine (AT)-rich sequences in all species (Figure 2A). The RSCU and frequency of amino acids were also analyzed, which revealed that leucine (Leu) is the most abundant, while cysteine (Cys) was the rarest amino acid in all four studied *Plantago* plastid genomes (Figure 2B). The codon usage also revealed a shift toward a higher number of codons having A/T at the third position (Figure 2C and Table S1).

### 2.2. Divergence Hotspots in Plantago Plastomes

Our comparison showed that all four studied *Plantago* chloroplast genomes had similar nucleotide compositions in all structural (LSC, SSC, and IR) and coding regions, which extended even to IGSs (Table S2). The number of substitutions ranged between 3119 (*P. atrata*) and 6358 (*P. maritima*), while substitution types were shared among species (Table 3). The most frequently occurring mutations were A/G and C/T conversions, compared with other SNPs (Table 3). 

The ratio of transitions (Ts) and transversions (Tv) in the plastid genomes ranged from 1.09 to 1.44 in the LSC and between 0.90 and 1.36 in the SSC, while varying from 0.8 to 1.22 in the IR region (Table S3). In general, the Ts were more frequent in four studied *Plantago* species, in line with observations in other plant species [27,39]. We found a huge difference in substitutions between the species of subgenera *Psyllium* (*P. argentea*, *P. atrata*, and *P. lanceolata*) and *Coronopus* (*P. maritima*). InDels were also examined, using DnaSP in all regions of the plastid genomes. The number of InDels ranged from 517 (*P. atrata*) to 687 (*P. maritima*). They were mostly located in the LSC and sparsely found in the SSC, whereas the IRs contained only a few InDels (Table 4). 

This may be because IRs are more conserved in plastid genomes and evolve under concerted evolution, while the LSC and SSC regions are more prone to substitutions [40]. We also found a difference in InDels specifically in the IR between the species of subgenera *Psyllium* (*P. argentea*, *P. atrata*, and *P. lanceolata*) and *Coronopus* (*P. maritima*). By considering all positions with single- or multinucleotide variations as SNPs, 16,247 SNPs were identified, corresponding to a mean SNP frequency of 16.247 SNPs/kb in *Plantago* species while InDels showed a mean frequency of 2.259/kb. Additionally, we also compared the three *Plantago* genomes that were already published for *P. lanceolata* (MW877582.1), *P. atrata* (MW877580.1), and *P. maritima* (KR297244.1) with our sequenced genomes. We found a significant difference in the number of SNPs and indels between the previously published genomes and our newly sequenced genomes (Table S4). The intraspecific diversity was further examined among the multiple accessions of *Plantago* plastomes for each species. *Plantago aristata* showed the lowest rate of substitutions per base (0.00001) indicating very low intraspecific diversity and *Plantago rigida* showed the highest rate of substitutions per base (0.00230), suggesting higher intraspecific diversity (Table S5; Figure S1). Furthermore, a total of 185 species-specific SNPs for *P. lanceolata* were also identified, which could serve as a basis for the design of molecular markers potentially able to distinguish *P. lanceolata* from other species within the *Plantago* genus (Table S6; see Section 2.7 below).

**Figure 3:**
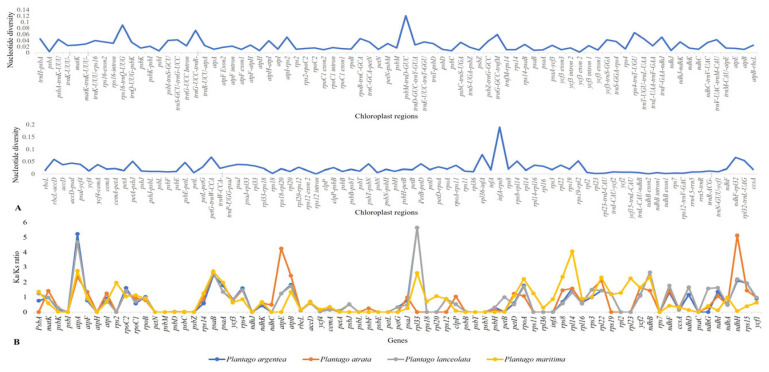
Polymorphism level and Ka/Ks ratios of different plastome regions: (**A**) Average π value for each coding and intergenic region of the 4 studied *Plantago* plastomes. (**B**) Ratio of Ka and Ks substitutions in 75 protein-coding genes of the plastomes of the four *Plantago* species.

The InDels and SNP mutational events in the plastid genome showed uneven distributions and clustered as ‘hotspots’ [41,42]. More polymorphism was shown in the IGS regions (average nucleotide diversity π = 0.0961) than in the gene’s regions (π = 0.0219) and intron (average nucleotide diversity π = 0.0155). Among the *Plantago* species, the values ranged from 0.0006 (*ndhB*) to 0.0432 (*ycf1* region) (Figure 3A). We selected 20 highly polymorphic regions for marker development that all belonged to IGS regions based on the analysis of mutation rates of the complete chloroplast genome sequences (Table 5). 

We further investigated the Ka and Ks substitutions and their ratio (Ka/Ks) by 75 protein-coding genes in the four studied *Plantago* species (Table S7 and Figure 3B).

The average Ka/Ks ratio for 75 protein genes analyzed in the four chloroplast genomes was 1.1118. There were significant differences in the evolutionary rates among these species; 19 of 75 protein genes had positively selected sites, which had Ka/Ks > 1, suggesting that these genes might have been subjected to positive selection during evolution. Especially four genes (*atpA*, *psaB*, *ndhB*, *ndhH*) had Ka/Ks ratios higher than 2.0 and these may be the most probable candidate genes for adaptive evolution. We selected these 19 genes for further analysis using BUSTED, FUBAR, and MEME. We found evidence of episodic diversifying selection only on the *rpoB* gene by using BUSTED. Next, we implemented FUBAR and MEME to detect rare sites that might be under positive selection. The results indicated that several codons exhibited evidence of positive selection in the following genes: *psaA*, *atpF*, and *rps4* (1 codon); *rpl33*, *rpoB*, *rps3*, and *atpB* (2 codons); and *rpl22* (8), *rpoC2*, and *ycf2* (9 codons) (Table S8). Most of the genes showed a relatively slow evolutionary divergence, indicating the conserved nature of the protein-coding genes that are found in the plastomes. Plastid genes are mostly subjected to purifying selection, and the low Ka/Ks ratio is due to the conservation of the functions of the photosynthetic apparatus.

### 2.3. Repeat Structure and Analyses

**Figure 4:**
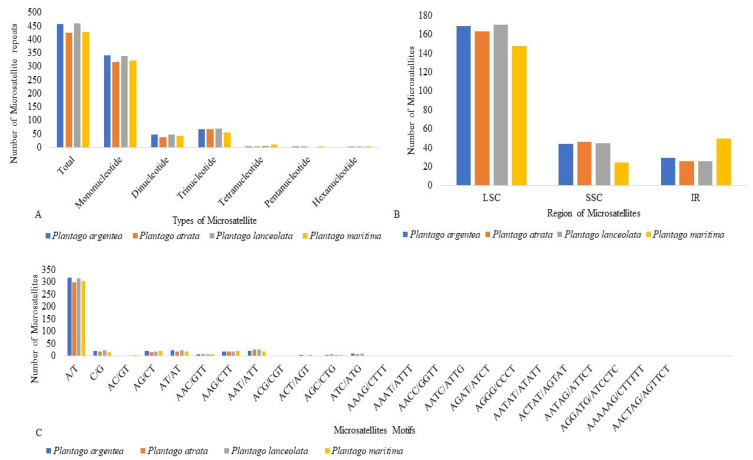
Comparative analysis of microsatellite repeats among four studied *Plantago* species: (**A**) Total number of microsatellites and their classification according to the number of repeat units. (**B**) The distribution of microsatellites among structural regions of the plastome. (**C**) Repeat unit composition of four studied *Plantago* microsatellites.

Repeats in the plastid genome are useful in evolutionary studies and play crucial roles in genome arrangement, plant breeding, and linkage map construction [43,44]. We performed a microsatellite analysis that revealed shared microsatellite loci ranging from 424 (*P. atrata*) to 459 (*P. lanceolata*). Most SSRs were mononucleotide stretches followed by trinucleotide and dinucleotide repeats. In these groups, A/T motifs were highly abundant in mononucleotides, and AT/TA motifs were frequently observed among dinucleotide SSRs. The mononucleotide SSR motifs varied from 7- to 17-unit repeats; in dinucleotide SSRs, the motif change was from 4- to 6-unit repeats, whereas other types of SSRs were present mainly in 3–5-unit repeats. Most SSRs occurred in the LSC, followed by SSC and IR (Figure 4; Table S9). 

**Figure 5:**
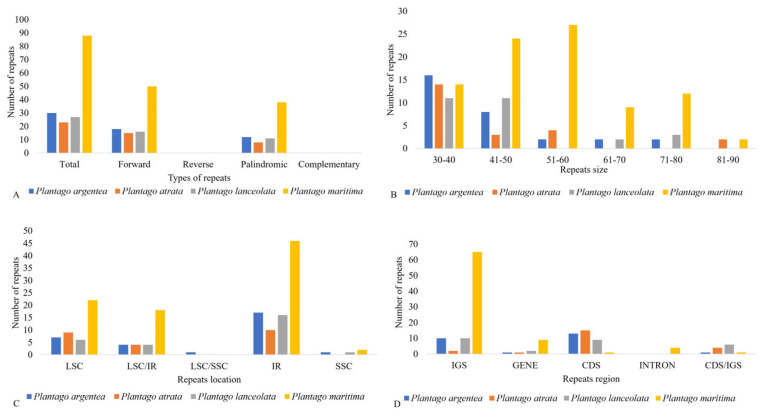
(**A**) Total number of oligonucleotides repeat among the four studied *Plantago* species and their distribution according to specific characteristics. (**B**) The distribution of repeats in size ranges. (**C**) The number of repeats grouped according to their location in each structural region. (**D**) The distribution of repeats in intergenic spacer regions (IGS), genes, coding DNA sequences (CDS), and introns and their proportionate occurrence.

We found slightly more SSRs in the IR of *P. maritima* (subgenus *Coronopus*) as compared to the species of the subgenus *Psyllium* probably due to the expansion of the IR. REPuter was also employed to locate further oligonucleotide repeats in all four studied *Plantago* species. Overall, 88 oligonucleotide repeats were found in *P. maritima* (subgenus *Coronopus*), which is significantly higher as compared to *P. argentea* (30), *P. atrata* (23), and *P. lanceolata* (27), all of which belongs to the subgenus *Psyllium*. The forward (F) and palindromic (P) repeats were present in all species, while no reverse (R) and complement (C) repeats were found in any of the *Plantago* plastomes analyzed. The oligonucleotide repeats were variable in size (30–90 bp) and a large fraction of the repeats were in the IR, with a net prevalence in the IGS for *P. maritima*, while with a more balanced distribution between CDS and IGS for the three species of the subgenus *Psyllium* (Figure 5 and Table S10). 

**Figure 6:**
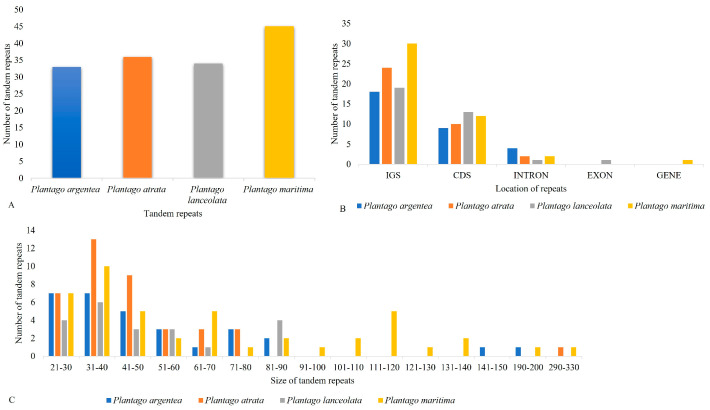
Assessment of tandem repeats: (**A**) Total number of tandem repeats and (**B**) their distribution among functional regions of the plastome. (**C**) Tandem repeat number, size, and distribution.

Moreover, 45 tandem repeats were detected in *P. maritima* (subgenus *Coronopus*), which is slightly higher as compared to *P. argentea* (33), *P. atrata* (36), and *P. lanceolata* (34), all of which belong to the subgenus *Psyllium*. The tandem repeats were variable in size (22–330 bp) and a large fraction of the repeats were in the IGS, followed by CDS and intronic regions (Figure 6 and Table S11).

### 2.4. Comparative Plastomics and Inverted Repeat Boundaries

**Figure 7:**
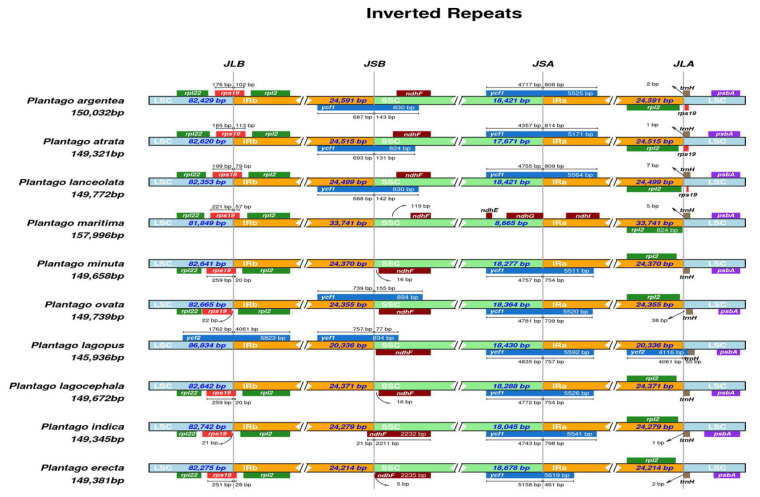
Schematic representation of junction sites in the plastomes of different *Plantago* species belonging to subgenera *Psyllium* and *Coronopus* (accession numbers listed in Table S12). The junction between LSC and IR is indicated as JL, while the junction between IR and SSC is indicated as JS. Genes above and below the different plastome regions are, respectively, in forward and reverse orientation. The number of bases in each region is reported for genes at the boundaries.

The plastid genome of land plants has a conserved quadripartite structure, but diversity exists at the junction sites of the major structural regions of the genome. The size range of LSC, SSC, and IR varies among the plastid genomes of the species, which may cause alterations in several genes, leading to deletion, duplication, or functional pseudogenization at the junction sites [45]. To investigate such events, we compared the JL (LSC/IR) and JS (IR/SSC) junction sites of 10 *Plantago* plastid genomes (Figure 7). 

The resemblance at junctions revealed the close relatedness among the three *Plantago* species of the subgenus *Psyllium*, where no obvious amplification or contraction events were detected. In *P. maritima* (subgenus *Coronopus*), however, the IRs significantly increased in size up to 9.15 kb as compared to other *Plantago* species of the subgenus *Psyllium*. Moreover, repeat expansion occurred in the SSC, resulting in the transfer of five former SSC genes (*ndhI*, *ndhA*, *ndhH*, *rps15*, *ycf1*) into the IR regions. Additionally, an important variation among the *Plantago* plastomes IR regions was a small-scale inversion related to the *ycf1* gene, which was detected only in the *P. maritima* plastome (Figure S2). 

The *rps*19 gene was found at the junction site of JLB (LSC/IRb), and a portion of this gene (20–113 bp) was copied in the IRa in all studied *Plantago* genomes except *P. ovata* and *Plantago indica*, while the *rpl2* gene was completely duplicated in IRs. The *ycf1* gene was situated at the IRb/SSC boundary, and a portion of this gene (77–155 bp) was copied in the SSC region. Still at this boundary, the *ndh*F gene was entirely present in the SSC region in all *Plantago* genomes except in *P. indica* (21 bp), where it extended across the IRb region. At the SSC/IRa boundary, the *ycf1* gene was consistently found in all studied *Plantago* plastid genomes and spanned 461–814 bp across the IRa boundary. The *rps19* pseudogene was exclusively identified in *P. argentea*, *P. atrata*, and *P. lanceolata*. 

### 2.5. Putative RNA-Editing Sites

RNA editing can modify the DNA-encoded sequencing of transcribed RNA by adding, deleting, or modifying the nucleotides [46]. RNA editing aids in creating transcripts and maintaining protein diversity [47]. To examine the RNA editing in four studied *Plantago* species, we predicted putative sites in the plastid genomes, using PREPACT, which revealed 67 putative sites in 10 genes of *P. atrata*, while 75, 76, and 95 editing sites were found in 11 genes of *P. argentea*, *P. lanceolata*, and *P. maritima*, respectively. 

Most of these RNA-editing sites were found in *rpoC1* (20), *rpcC2* (16), and *ndh*A (12). All four studied *Plantago* species had high levels of conversion from threonine (Thr) to isoleucine (Ile) [22.6% (*P. argentea)*, 20.8% (*P. atrata)*, and 23.6% (*P. lanceolata)*], followed by proline (Pro) to serine (Ser) [14.28%, 17.94%, and 16.21%, respectively]. By contrast, in *P. maritima* high levels of conversion for proline (Pro) to leucine (Leu) (22.1%) were found followed by proline (Pro) to serine (Ser) (16.8%). Out of all the putative RNA-editing sites detected 52 (69.3%), 48 (71.6%), 51 (67.1%), and 61 (64.2%) codons substituted on the second nucleotide in *P. argentea*, *P. atrata*, *P. lanceolata*, and *P. maritima*, respectively. Many amino acids were converted from Thr to Ile and changes at these sites assisted in the formation of hydrophobic amino acids, e.g., valine (Val), leucine (Leu), and phenylalanine (Phe) (Table S13). 

### 2.6. Phylogenetic Analysis

**Figure 8:**
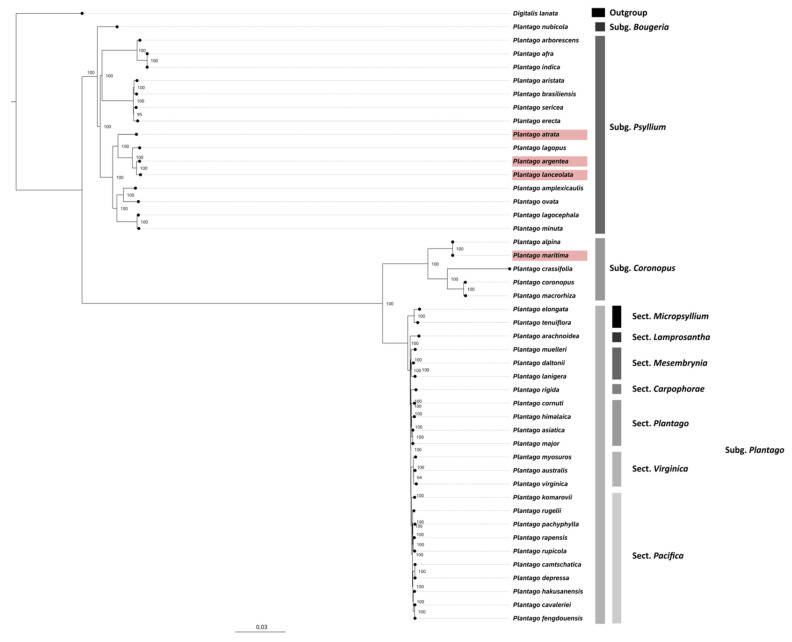
Maximum likelihood phylogenetic reconstruction of 45 *Plantago* species based on fully sequenced plastomes.

Maximum likelihood phylogenetic reconstruction for 45 *Plantago* species was carried out based on alignments of complete chloroplast genomes (accession numbers listed in Table S13). The analysis was based on a 213,372 bp alignment using the best-fit model TVM + F + R7 and resulted in a phylogenetic tree supported by high bootstrap values (Figure 8). 

Among the species we sequenced, *P. maritima* was a more divergent species as compared to *P. atrata*, *P. argentea*, and *P. lanceolata*. The latter two species were very closely related and formed a single clade with high bootstrap (100%) support. The whole-plastome tree topology obtained here also indicated that the *Plantago* genus is monophyletic with strong support (BS = 100%) and the subgenus *Bougueria* is sister to the remainder subgenera of *Plantago*. Moreover, within the subgenus, *Plantago* sect. *Micropsyllium* is sister to the remainder of subg. *Plantago* (BS = 100%). The divergence times of *Plantago* species analyzed in the current study were estimated using a relaxed uncorrelated clock implemented in BEAST. The results show that the divergence of *Aragoa* and *Plantago* occurred about 8.43 million years ago (Mya) in the Late Miocene, and the diversification of the subgenus *Psyllium* and subgenus *Coronopus* occurred at 3.62 and 3.38 Mya, respectively. Furthermore, *P. argentea* and *P. lanceolata* began to differentiate from the common ancestor into two distinct lineages at 0.3 Mya (Figure S3).

### 2.7. Development of a P. lanceolata-Specific Molecular Marker

**Figure 9:**
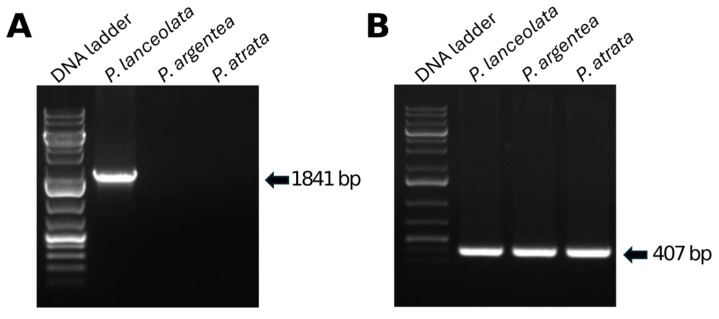
Amplification patterns with plastome markers: (**A**) The amplicon obtained with primer combination PlaLan_1F + PlaLan_2F is specific for *P. lanceolata*. (**B**) The amplicon obtained with the Pla_CTRL_F + Pla_CTRL_R primer combination amplifies from three *Plantago* species.

Given the relevance of *P. lanceolata* for the herbal market and its cosmopolitan distribution, we set out to develop a PCR assay that could reliably discriminate this plantain species from all the others for which whole-plastome sequences are available. Due to the close phylogenetic relatedness, as well as the very high sequence similarity of *P. argentea*, *P. atrata*, *P. lagopus*, and *P. lanceolata*, we did not find strong candidates among the top 20 mutational hotspots identified above for the *Plantago* genus. We, therefore, manually curated the design of primer pairs in the regions with nucleotide polymorphisms distinguishing the *P. lanceolata* plastome from all other published *Plantago* plastomes. This resulted in a primer pair (PlaLan_1F + PlaLan_2F) whose forward primer was designed in the *rpoC2* CDS, and the reverse primer in the *rpoC2-rpoC1* IGS (Table S14). Each primer 3’ end corresponds to a *P. lanceolata*-specific SNP (Table S14; Figure 9). In addition, a control primer pair (Pla_CTRL_F + Pla_CTRL_R) was designed in a region of the *rbcL* gene, which is fully conserved among *Plantago* species. In addition to *P. lanceolata*, the genomic DNA of *P. atrata* and *P. argentea* were used as templates in PCR reactions using either primer combination. According to expectations, a strong amplification band of about 1850 bp was obtained specifically for *P. lanceolata*, but not for *P. atrata* and *P. argentea*. On the other hand, the positive control amplification provided a single band of about 400 bp for all three species (Figure 9), demonstrating that the lack of amplification in *P. atrata* and *P. argentea* is not due to poor DNA quality. Taken together, these results confirm that the PlaLan_1F + PlaLan_2F primer combination reliably discriminates *P. lanceolata* from its closest relatives.

## 3. Discussion

Whole-plastome sequencing has revolutionized many fundamental and applied fields of plant biology. In this study, we applied whole-plastome sequencing of four accessions of *Plantago* to support the development of species-specific molecular markers for *P. lanceolata*. From an applicative point of view, in fact, the complete chloroplast genome has been proposed as a potential candidate for the next generation of DNA barcodes in plants [38]. Previously, it was demonstrated that the identification of highly variable regions by comparative plastomics could provide insights into the loci that could be used in DNA barcoding [39,40]. The hotspot regions identified in this study could in principle be useful in such DNA barcoding investigations at the generic level for species identification [41,42]. The extreme phylogenetic relatedness and sequence identity of *P. lanceolata*, especially to *P. argentea*, *P. atrata*, and *P. lagopus*, however, made arduous the development of a suitable barcoding marker even from these 20 mutational hotspot regions. By leveraging on the availability of the whole plastome for a high number of *Plantago* species and of multiple plastomes for *P. lanceolata* as well as some other species, however, we could develop a highly selective primer in the *rpoC1-rpoC2* region, which, despite being less polymorphic, hosted two SNPs that are highly diagnostic for *P. lanceolata*. While intraspecific variation in plastome sequences is known to occur [43,44,45], a minority of studies have included it as a validation of the markers designed. This is particularly relevant for the *Plantago* genus, which has accelerated plastome evolution [46]. Indeed, while the accessions of *P. lanceolata*, *P. atrata*, and *P. maritima* that we re-sequenced in our study, as expected, clustered with high support with conspecific sequences previously published, they show substitution levels in the range observed for other *Plantago* species (Table S5). However, none of the intraspecific substitutions affected the region selected for primer design, thus further confirming the robustness of the developed marker for the identification of the target species. These results highlight the importance of tailoring the design of barcoding primers to the specific set of species and the question at hand, as mutational hotspots may not be providing optimal discrimination power for very closely related species. 

The comparative analyses of newly sequenced accessions of *P. lanceolata*, *P. atrata*, and *P. maritima*, as well as the novel plastome of *P. argentea*, with previously published sequences, confirmed most of the general features already determined for *Plantago* plastomes. In particular, the plastomes of many land plant lineages are evolving quite slowly in terms of sequence and structural organization compared to their mitochondrial or nuclear counterparts [47,48]. Among the hotspots for structural reorganizations inside plastomes are the IRs, which regularly are subject to expansion, contraction, or even complete loss. Such modifications have occurred independently numerous times throughout land plant evolution, frequently specific to orders and families, occasionally even to one or a few species within a genus [49,50]. In *Plantago*, however, we confirmed that the plastomes from the subgenus *Coronopus* show significant genomic change, including increased genome size, gene content, repeat structures, substitutions and indels, RNA-editing sites, and expansions and inversions of the inverted repeat that simultaneously reduced the size of the SSC as compared to the subgenus *Psyllium*. The size of subgenus *Coronopus* plastomes currently available in GenBank varied from 157 to 159 kb as compared to the subgenus *Psyllium* (145–150 kb). Similarly, the IR size of subgenus *Coronopus* plastomes varied from 33 to 34 kb as compared to the subgenus *Psyllium* (20–24 kb). The number of genes also varied between the currently available plastomes in the GenBank belonging to the subgenus *Coronopus* (137–138) as compared to the subgenus *Psyllium* (127–133). Furthermore, in *P. maritima* (subgenus *Coronopus*), a unique gain of a *trnL-CAG* gene via duplication and the diversification of the more common plastid gene *trnL-UAG* were observed. In *P. maritima* (subgenus *Coronopus*), additional synapomorphies include losses of the *rpl2* and *clpP* introns. Additionally, we observed that in the subgenus *Psyllium*, there were 18 (with 12 protein-coding and 6 tRNA genes) intron-containing genes as compared to the subgenus *Coronopus* where the number was 16 (with 10 protein-coding and 6 tRNA genes). Similar changes were also observed in other species of *Plantago* [13,51] and other genera such as *Silene* [52] and *Passiflora* [53]. Generally, these chloroplast characteristics and structural organizational variations provide strong complementary support for the phylogenetic trees produced from the whole-plastid nucleotide sequence, which are in line with the results reported by Mower et al., 2021 [13], regarding (1) the reassignment of *Bougueria nubicola* as a member of *Plantago* (as *Plantago nubicola*), which had been indicated (but with weak support) in previous studies [2,12,54]; and (2) a sister-group relationship between a monophyletic subg. *Coronopus* and a monophyletic subg. *Plantago*, a result that in previous studies had been either weakly recovered [12,55,56,57] or weakly contradicted [54,58]. Additionally, our phylogenomic analyses also showed with full support the close relatedness of *P. argentea* to *P. lanceolata*, thus confirming the weakly supported relationship identified earlier with a limited number of markers [5].

Plants have evolved complex physiological and biochemical adaptations to adjust and adapt to different environmental stresses. We analyzed the patterns of synonymous (Ks) and non-synonymous (Ka) substitution of protein-coding genes, which are essential markers in evolutionary genetics for defining slow- and fast-evolving genes [59]. While substitution rate variations have been previously associated in comparative studies of *Plantago* and Plantaginaceae with plastome structural changes like inversions and IR expansions/contractions [13,46], to the best of our knowledge, no detailed analyses on the pattern of adaptive evolution have been carried out on plastidial genes of the genus. The Ka/Ks ratios in our analysis indicate, for the first time, signatures of changes in selective pressures in a total of 10 plastome genes, which had Ka/Ks values greater than one, possibly due to either relaxed purifying selection, increased positive selection, or a combination of the two. This finding was conclusively supported by an integrative analysis using the Fast Unconstrained Bayesian AppRoximation (FUBAR) and Mixed-Effects Model of Evolution (MEME) methods, which identified a specific set of positively selected codons within these genes. Among these genes, two are involved in chloroplast transcription (*rpoB*, *rpoC2*), four in chloroplast translation (*rps3*, *rps4*, *rpl22*, and *rpl33*), one in protein import (*ycf2*), and three in energy generation through photosynthesis (*psaA*, *atpB*, and *atpF*). Chloroplast gene expression has recently emerged as an important factor in plant responses to environmental stresses, ranging from chilling to high light, salinity, osmotic, and high-temperature stress [60]. Our results show that both phases of chloroplast gene expression, transcription, and translation, show signs of positive selection in the studied *Plantago* species: rpoB and rpoC2 are two of the four plastid-encoded RNA polymerase (PEP) core subunits [61], while the ribosomal genes putatively under positive selection belong to both the small and the large subunit of the chloroplast ribosome [62]. Both *rpoB* and *rpoC2* were among the five positively selected plastome genes in the genus *Hosta* [63], suggesting that they could be implicated in climate change adaptation of the species that underwent rapid diversification in East Asia during the Miocene [64]. Based on previous results and in agreement with the molecular dating reported in this work [12] (Figure S3), during the late Miocene, the *Plantago* genus also underwent a diversification phase associated with the split between the clades encompassing the subgenera *Plantago*/*Coronopus* and *Psyllium*/*Bougueria*. It is, therefore, possible that the complex climatic oscillations that took place in the late Miocene and the beginning of the Pliocene [65,66] could have at least in part contributed to determining the selective patterns observed in *rpoB* and *rpoC2* genes of the studied *Plantago*. Among the ribosomal genes, knockout of *rpl33* has been shown to increase plant sensitivity specifically to cold stress, but not to heat or to low or high light [67], suggesting that this gene may have contributed to the adaptation of the studied *Plantago* species to the glacial phases of the Pleistocene glaciation, consistent with the dating results indicating that after about 3.5 MA, a second phase of *Plantago* subgeneric differentiation and speciation took place in both the major clades mentioned above. Contrary to former assumptions, in fact, recent molecular evidence suggests that Quaternary climatic oscillations probably were important drivers of many plant speciations and radiations [68]. Although not directly involved in chloroplast protein expression, ycf2, involved in the ATP-driven import of nuclear proteins through the inner chloroplast membrane [69], contributes to the correct functioning of chloroplast protein complexes. Like in the studied *Plantago*, *ycf2* has been found to bear signatures of positive selection in several other genera [70,71,72,73]. Among the photosynthetic genes, atpF, a subunit of the plastidial H+-ATP synthase responsible for electron transport and photophosphorylation during photosynthesis [74], has been associated with the adaptation to cold/drought stress in the *Quercus* species [75]. Intriguingly, psaA, a core subunit of photosystem I (PSI), has been associated with the adaptation to different light intensities in the genus *Oryza* [76] and PSI is known to play a relevant role in protection from photoinhibition by regulating photosynthetic cyclic electron flow [77]. As light stress is a common consequence of other types of stress impairing photosynthesis, like light, low temperature, and drought [77], the repeated cold spells and the cycles of aridification of the climate during the late Miocene and throughout the Pleistocene discussed above may also have been among the causes of the positive selective signatures in the *psaA*, *atpB*, and *atpF* genes of the studied *Plantago* species.

## 4. Materials and Methods

### 4.1. Plant Materials, DNA Extraction, and Illumina Sequencing

Fresh lush green leaves of *Plantago* species were collected from the Trentino region, Italy (46.0666° N, 11.1166° E). The leaf samples were rinsed with 70% ethanol, and total genomic DNA was extracted with DNeasy Plant Mini Kit (Qiagen, Hilden, Germany). DNA quality and concentration were assessed by gel electrophoresis on a 1% agarose gel and using the Qubit 4 Fluorometer (Thermo Fisher Scientific, Waltham, MA, USA), respectively. A total of 300 ng of extracted genomic DNA for each species was independently sheared with Covaris S220 (Covaris Inc., Woburn, MA, USA) to the average size of 400 bp and used for Illumina-sequencing library preparation. Each library was prepared with TruSeq DNA sample preparation kits for paired-end sequencing (Illumina Inc., San Diego, CA, USA) similarly as previously described [74], pooled in equimolar ratio, and sequenced on an Illumina HiSeq2000 sequencer (CIBIO NGS Facility, Povo (TN), Italy).

### 4.2. Assembly, Annotation, and Visualization of Plantago Species Plastomes

The bowtie program [78] was used to identify and remove phiX174 contaminants using the parameters −t −p 10 −un −S. The raw sequencing read quality was checked with the FastQC v0.12.0 tool [79]. The reads were trimmed with FASTX-Toolkit v0.0.14 (http://hannonlab.cshl.edu/fastx_toolkit/; accessed on 16 April 2024), with parameters –q 20 –p 90 –Q 33, and adapters were eliminated by Trimmomatic v0.32 [80] using PE -phred33 ILLUMINACLIP: TruSeq3-PE-2.fa:2:30:10 LEADING: 3 TRAILING: 3 SLIDINGWINDOW: 4:15 MINLEN: 100 as settings. Then, FastQC was used once again to carry out the final reads quality check with default parameters. GetOrganelle 1.7.7.1 [81] was used for assembly using the parameters −w 48 −R 25 −k 21,45,65,85. Gene annotation was conducted using GeSeq [82] with a BLAT [83] search using 85% identity to annotate protein-coding genes, rRNAs, and tRNAs. CPGAVAS2 [84] was used with default parameters by selecting option 1 “43-plastome”. After automatic annotation, start/stop codons and the position of introns were further confirmed manually by visual inspection of the translated protein of each gene in Geneious R9.0.5 (http://www.geneious.com) and BLAST search using default settings with homologous genes of plastid genomes of Plantaginaceae. The tRNA genes were further verified by tRNAscan-SE v2.0 [85] with default settings using options: sequence source “Organellar tRNA”, search mode “Default”, genetic code “Universal”, and Cut-off score for reporting tRNAs “15”. ARAGORN v1.2.38 [86] was used with default parameters by selecting genetic code “Bacterial/Plant chloroplast” with maximum intron length of 3000 bp. Circular genome maps were drawn with Chloroplot software [84] (https://irscope.shinyapps.io/Chloroplot/; accessed on 16 April 2024) by uploading the GenBank (.gb) format. The average coverage depth of *Plantago* species plastid genomes was calculated with GetOrganelle 1.7.7.1 [81]. The novel *Plantago* plastid genomes were deposited in the National Center for Biotechnology Information (NCBI) under the following accession numbers: *P. argentea* (PP541855), *P. atrata* (PP541856), *P. lanceolata* (PP541857), and *P. maritima* (PP5541858).

### 4.3. Structural Features, Codon Usage, and Amino Acids Frequencies

Codon usage and amino-acid frequencies were analyzed by using Geneious R9.0.5 software (http://www.geneious.com). The intergeneric comparison was carried out to gain insight into differences and syntenies that may exist between *Plantago* species. Circoletto [87] was used to compare structural features of *Plantago* chloroplast genomes using blastn search (e-value of <1 × 10^−10^) to create a Circos output.

### 4.4. SNPs, Indel, and Mutational Hotspots Detection with PCR Primer Designing

All de novo chloroplast genomes were aligned with multiple alignments using fast Fourier transform (MAFFT) 7.309 [88], using default parameters. Protein-encoding genes, intergenic spacer (IGS) regions, and introns were extracted to calculate the average number of nucleotide differences per site or nucleotide diversity (π) with a 100 bp window size as implemented in DnaSP v6 [89]. The substitution, transition (Ts), and transversion (Tv) rates were resolved from the MAFFT alignment, using *Plantago ovata* as a reference. Each structural element, including the LSC, SSC, and IR, was aligned individually to analyze SNPs and indel polymorphisms with Geneious and DnaSP, respectively. Furthermore, MEGA 11 [90] software was utilized to assess the intraspecific diversity of the complete chloroplast genome of *Plantago* species. Specific primers were designed with Primer3 [91], focusing on the conserved nucleotide sequences at both ends of mutational hotspots. 

### 4.5. IRs Junction Positions and RNA Editing Sites Prediction

The junction sites of the IRs and their border positions were compared using all *Plantago* species using the default setting of the IRscope [92]. The Plant RNA Editing Prediction & Analysis Computer Tool (PREPACT) [93] was used to predict putative RNA editing sites using default settings with *Plantago ovata* as a reference.

### 4.6. Microsatellites, Oligonucleotide, and Tandem Repeat Analysis

Microsatellite repeats within the chloroplast genomes of *Plantago* species were detected by the MIcroSAtellite identification tool (MISA) [94] with the minimal repeat number: 7 for mononucleotide SSRs, 4 repeat units for dinucleotide SSRs, and 3 repeat units for tri-, tetra-, penta-, and hexanucleotide SSRs. The REPuter software [95] (https://bibiserv.cebitec.uni-bielefeld.de/reputer; accessed on 16 April 2024) with minimal repeats size set to 30 bp, hamming distance to 3, minimum similarity percentage of two repeats copies up to 90%, and maximum computed repeats numbers to 500 bp parameters was used for scanning and visualizing genomic repeats including forward (F), reverse (R), complementary (C), and palindromic (P) repeats. Tandem repeats were found using the Tandem Repeats Finder v4.09.1 [96] with default parameters. 

### 4.7. Selective Pressure Analysis of Plantago Plastomes

The ratios of synonymous (Ks) and non-synonymous (Ka) substitutions for each extracted protein-encoding gene were calculated with DnaSP for all *Plantago* species, using *Plantago ovata* as a reference. The data were interpreted as Ka/Ks > 1, Ka/Ks = 1, and Ka/Ks < 1, representing positive, neutral, and purifying selection, respectively. Furthermore, we evaluated the impact of positive selection using additional codon models to estimate the rates of synonymous and nonsynonymous substitution. The signs of positive selection were further assessed using fast unconstrained Bayesian approximation (FUBAR) [97], the mixed effects model of evolution (MEME) [98] and (BUSTED) [99] as implemented in the DATAMONKEY web server [100]. Sites with cut-off values of PP > 0.9 in FUBAR were considered candidates to have evolved under positive selection. From all the analyses performed in DATAMONKEY, the most suited model of evolution for each dataset was selected as directly estimated on this web server. In addition, the mixed effects model of evolution (MEME), a branch-site method incorporated in the DATAMONKEY server, was used to test for both pervasive and episodic diversifying selection. MEME applies models with variable *ω* across lineages at individual sites, restricting *ω* to ≤1 in a proportion p of branches and unrestricted at a proportion (1 − p) of branches per site.

### 4.8. Plastid Phylogenomic Analysis

We included all available *Plantago* plastid genome sequences in our analysis (Organelle Genome Resources of NCBI, accessed on 16 April 2024). We used *Digitalis lanata* Ehrh. as an outgroup to root our tree from the closet tribe Digitalideae to understand the evolutionary relationships within the genus. For phylogenetic analysis, the complete chloroplast genomes were aligned using MAFFT, and then tree searches were performed in IQ-TREE 1.5.5 [101]. We used IQ-TREE to infer the best-fitting models of substitution for partitioning the matrix-combining multiple genes with the -TESTMERGEONLY and -AICc (Akaike information criterion corrected for small sample sizes) options in the built-in ModelFinder [102]. Maximum likelihood (ML) analyses were performed, using the ultrafast bootstrap approximation (UFBoot; [103] with 1000 replicates. The key idea behind UFBoot is to keep trees encountered during the ML-tree search for the original sequence alignment and to use them to evaluate the tree likelihoods for the bootstrap sequence alignment. UFBoot provides relatively unbiased bootstrap estimates under mild model misspecifications and reduces computing time while achieving more unbiased branch support than standard bootstrap [103]. The SH-like approximate likelihood ratio test (SH-aLRT) was also conducted together with UFBoot, while iTOL [104,105,106] was used for the visualization of a phylogenetic tree.

Relative divergence times were estimated for *Plantago* species analyzed in the current study by using BEAST v.1.10.4 [107], applying GTR + I + G rate substitution to the protein-coding plastid gene matrix. A birth and death tree prior and an uncorrelated relaxed clock model that allows rates to vary independently along branches were used [108] with all other parameters set to default. The median time split between *Plantago* and *Aragoa* species (mean = 7.1 Myr; standard deviation = 1.0) was used as a temporal constraint to calibrate the BEAST analyses derived from Rønsted et al. (2002) [12]. Uncertainty regarding this date was incorporated by assigning normal prior distributions to the calibration point [109,110]. Four independent BEAST runs were conducted with Markov Chain Monte Carlo (MCMC) samples based on 100 million generations, sampling every 1000 generations. Convergence of all parameters was assessed in Tracer 1.5 [111] and 10% of each chain was removed as burn-in. The Markov chains were combined in LogCombiner 1.7.2. [107] to calculate the maximum clade credibility tree.

## 5. Conclusions

The taxonomy of *Plantago* is notoriously difficult, mainly because its low morphological variation, reduced morphology, and lack of useful taxonomic characters have prevented a full understanding of the evolution and taxonomic delimitation of the genus and its species [11,12]. In these complicated groups, well-known plastid barcode regions (e.g., *trnH*-*psbA*, *matK*) might not have sufficient polymorphism and thus may not be able to provide species-specific information necessary for differentiation [112]. It has been shown that plastome-based “super barcoding” could overcome these difficulties and could differentiate species in difficult taxonomic groups [113]. Here, we investigated whether ‘super-barcoding’ based on plastid genomes could be applied to closely related *Plantago* species by comparing the complete plastome sequences of four species, among which the one of *P. argentea* was obtained for the first time. By comparing our sequences to previously published plastomes, we further identified sequence divergence hotspots and located repeat sequences and indels in the plastomes of *Plantago* species. These regions may provide a useful means of developing suitable molecular markers for species identification and DNA barcoding of *Plantago* medicinal products, although in the case of closely related species belonging to particular clades the use of diagnostic SNPs in less polymorphic regions may be very valuable. Hopefully, by demonstrating the usefulness of species-specific SNPs, our study will provide a solid basis for future studies and assist in the development of DNA barcoding markers for the clarification of the taxonomic identity of *Plantago* species in medicinal plant production. Such plastome-based “super barcoding” could be repeatable, reliable, and sensitive enough to discriminate similar *Plantago* species, as we experimentally demonstrated in the case of *P. lanceolata*.

## Figures and Tables

**Table 1:** Comparison and general features of chloroplast genomes of *P. argentea*, *P. atrata*, *P. lanceolata*, and *P. maritima*.

Characteristics		*P. argentea*	*P. atrata*	*P. lanceolata*	*P. maritima*
Accession No.		PP541855	PP541856	PP541857	PP5541858
Coverage depth		103×	50×	160×	80×
Size (bp)		150,092	149,381	149,832	158,056
LSC length (bp)		82,489	82,680	82,413	81,909
SSC length (bp)		18,421	17,671	18,421	8665
IR length (bp)		24,591	24,515	24,499	33,741
Number of genes		133	133	133	137
Protein-coding genes		86	86	86	90
tRNA genes		38	38	38	39
rRNA genes		8	8	8	8
Duplicate genes		18	18	18	22
GC content	Total (%)	38.3%	38.3%	38.4%	38.6%
	LSC (%)	36.5%	36.5%	36.6%	37%
	SSC (%)	32.5%	32.3%	32.6%	32.8%
	IR (%)	43.5%	43.5%	43.6%	41.4%
	CDS (%)	38.5%	38.5%	38.5%	38.7%
	rRNA (%)	55.2%	55.2%	55.2%	55.2%
	tRNA (%)	52.8%	52.8%	52.9%	53.8%
	All gene %	40.2%	40.2%	40.2%	40.4%
Protein coding part (CDS) (%bp)		51.7%	51.6%	51.7%	53.9%
All gene (%bp)		72.5%	72.4%	72.6%	73%
Non-coding region (%bp)		27.5%	27.6%	27.4%	27%

**Table 2:** Genes of chloroplast genomes of *P. argentea*, *P. atrata*, *P. lanceolata*, and *P. maritima*.

Category for Gene	Group of Gene	Name of Gene				
Photosynthesis-related genes	Photosystem I	*psaA*	*psaB*	*psaC*	*psaI*	*psaJ*
	Photosystem II	*psbA*	*PsbB*	*psbC*	*psbD*	*psbE*
		*psbF*	*psbH*	*psbI*	*psbJ*	*psbK*
		*psbL*	*psbM*	*psbN*	*psbT*	*psbZ*
	Cytochrome b/f complex	*petN*	*petA*	*petL*	*petG*	*petB* *
		*petD* *				
	ATP synthase	*atpI*	*atpH*	*atpA*	*atpF* *	*atpE*
		*atpB*				
	Assembly/stability of photosystem I	*ycf3* **	*ycf4*			
	NADPH dehydrogenase	*ndhB* *^,a^	*ndhH*	*ndhA* *	*ndhI*	*ndhG*
		*ndhJ*	*ndhE*	*ndhF*	*ndhC*	*ndhK*
		*ndhD*				
	Rubisco	*rbcL*				
Transcription- and translation- related genes RNA genes	Transcription Small subunit of ribosome	*rpoA*	*rpoC2*	*rpoC1* *	*rpoB*	*rps16* *
		*rps7* ^a^	*rps15*	*rps19*	*rps3*	*rps8*
		*rps14*	*rps11*	*rps12* ^a,^*	*rps18*	*rps4*
		*rps2*				
	Large subunit of ribosome	*rpl2* ^a,^*	*rpl23* ^a^	*rpl32*	*rpl22*	*rpl14*
		*rpl33*	*rpl36*	*rpl20*	*rpl16* *	
	Ribosomal RNA	*rrn16* ^a^	*rrn4.5* ^a^	*rrn5* ^a^	*rrn23* ^a^	
	Translation initiation factor	*infA*				
	Transfer RNA	*trnV-GAC* ^a^	*trnI-CAU* ^a^	*trnA-UGC* ^a,^*	*trnN-GUU* ^a^	*trnP-UGG*
		*trnW-CCA*	*trnV-UAC* *	*trnL-UAA* *	*trnF-GAA*	*trnR-ACG* ^a^
		*trnT-UGU*	*trnG-UCC* *	*trnT-GGU*	*trnR-UCU*	*trnE-UUC*
		*trnY-GUA*	*trnD-GUC*	*trnC-GCA*	*trnS-GCU*	*trnH-GUG*
		*trnK-UUU* *	*trnQ-UUG*	*trnfM-CAU*	*trnG-GCC*	*trnS-UGA*
		*trnS -GGA*	*trnF-GAA*	*trnM-CAU*	*trnL-CAA* ^a^	*trnL-UAG*
		*trnI-GAU* *^,a^				
Other genes	RNA processing	*matK*				
	Carbon metabolism	*cemA*				
	Fatty acid synthesis	*accD*				
	Proteolysis	*clpP* *				
	C-type cytochrome synthesis gene	*ccsA*				
	Component of TIC complex	*ycf1* ^a^				
	Hypothetical proteins	*ycf2* ^a^				

* Gene with one intron, ** Gene with two introns, ^a^ Gene with two copies.

**Table 3:** Comparison of substitutions in *Plantago* species.

Types	*P. argentea*	*P. atrata*	*P. lanceolata*	*P. maritima*
A/G	954	888	983	1650
C/T	998	924	1002	1616
A/C	374	329	369	897
C/G	207	158	204	506
G/T	410	389	407	980
A/T	434	431	428	709
**Total**	3377	3119	3393	6358
**LSC**	2526	2422	2538	5127
**SSC**	720	572	713	616
**IR**	131	125	142	615

*Plantago ovata* was used as a reference for SNP detection.

**Table 4:** Distribution of InDels (per bp) in *Plantago* chloroplast genomes.

	*P. argentea*	InDel Sites (bp)	InDel Average Length
LSC	411	4490	10.92
SSC	73	577	7.90
IR	39	410	10.51
	***P. atrata***	**InDel sites (bp)**	**InDel average length**
LSC	419	4271	10.19
SSC	66	1181	17.89
IR	32	346	10.81
	***P. lanceolata***	**InDel sites (bp)**	**InDel average length**
LSC	416	4366	10.49
SSC	78	607	7.782
IR	38	420	11.05
	***P. maritima***	**InDel sites (bp)**	**InDel average length**
LSC	580	9090	15.67
SSC	50	10,263	205.26
IR	57	2269	39.80

*P. ovata* was used as a reference for InDel detection.

**Table 5:** Mutational hotspots among *Plantago* species.

ID	Region	Nucleotide Diversity	InDel Diversity	No. of Mutation	Region Length
1	*infA-rps8*	0.18967	0.00955	230	333
2	*psbM-trnD-GUC*	0.12037	0.00361	91	1059
3	*rps16-trnQ-UUG*	0.08976	0.0119	117	224
4	*rpl36-infA*	0.07831	0.01124	12	246
5	*trnG-UCC-trnR-UCU*	0.07237	0.0122	21	1584
6	*ndhD-psaC*	0.06915	0.0101	13	636
7	*petG-trnW-CCA*	0.0678	0.0056	14	345
8	*ndhF-rpl32*	0.06681	0.00564	84	226
9	*rps4-trnT-UGU*	0.06469	0.00918	34	814
10	*trnG-GCC-trnfM*	0.05894	0.00877	18	713
11	*rbcL-accD*	0.05836	0.00534	108	356
12	*rpl32-trnL-UAG*	0.05348	0.00514	58	183
13	*rps19-rpl2*	0.05238	0	6	333
14	*rps8-rpl14*	0.05121	0.0036	16	111
15	*atpI-rps2*	0.05109	0	20	120
16	*trnF-GAA-ndhJ*	0.051	0.00711	28	765
17	*petA-psbJ*	0.05045	0.0106	78	691
18	*rpoB-trnC-GCA*	0.04623	0.0078	89	195
19	*trnT-UGU-trnL-UAA*	0.04614	0.00638	61	107
20	*psbE-petL*	0.04558	0.00654	63	1524

## Data Availability

The original contributions presented in the study are included in the article/Supplementary Material, further inquiries can be directed to the corresponding author/s.

## Supplementary Materials

The following supporting information can be downloaded at: https://www.mdpi.com/article/10.3390/plants13192691/s1, Figure S1: Multiple accessions phylogeny of our sequenced *Plantago* genomes with already published one; Figure S2: Small-scale inversion related to *ycf1* gene in the IR of the *P. maritima* chloroplast genome by using (A) OGDRAW and (B) Mauve alignment; Figure S3: Divergence time estimation and diversification of *Plantago* species using *Aragoa abietina* as an outgroup; Table S1: Codon usage in the chloroplast genome of *P. argentea*, *P. atrata*, *P. lanceolata*, and *P. maritima*; Table S2: Base composition in the chloroplast genome of *P. argentea*, *P. atrata*, *P. lanceolata*, and *P. maritima*; Table S3: The distribution and characteristics of SNPs found among *Plantago* plastid genomes; Table S4: Comparison of SNPs and Indels of our sequenced and already published *Plantago* species plastomes; Table S5: Intraspecific diversity between the multiple accession *Plantago* plastomes; Table S6: *P. lanceolata* species-specific SNPs; Table S7: Comparison of synonymous and non-synonymous substitution in *Plantago* species; Table S8: Positively selected sites in protein-coding genes and list of converted amino acids by using BUSTED, FUBAR, and MEME in *Plantago* species; Table S9: Microsatellites loci in the chloroplast genome of *P. argentea*, *P. atrata*, *P. lanceolata*, and *P. maritima*; Table S10: Oligonucleotide repeats in chloroplast genomes of *P. argentea*, *P. atrata*, *P. lanceolata*, and *P. maritima*; Table S11: Tandem Repeat sequences in the chloroplast genome of *P. argentea*, *P. atrata*, *P. lanceolata*, and *P. maritima*; Table S12: NCBI GenBank accession numbers used in this study; Table S13: Putative RNA editing sites composition in the chloroplast genome of *P. argentea*, *P. atrata*, *P. lanceolata*, and *P. maritima*; Table S14: Barcoding primers developed in this study.
