# Supplementary material for: Comparative Plastomics of Plantains (Plantago, Plantaginaceae) as a Tool for the Development of Species-Specific DNA Barcodes
Source: Plants (Basel). 2024 Sep 25;13(19):2691. doi: 10.3390/plants13192691 (PMC11478842; doi:10.3390/plants13192691)
Supplement: Supplementary file 1 [file plants-13-02691-s001.zip › plants-3143619-supplementary/Table S11. Tandem Repeats_01.docx]

**Table S11**. Tandem Repeat sequences in the *Plantago argentea* chloroplast genome.

| **Serial no** | **Indices** | **Repeat**  **Length** | **Size of repeat unit × Copy number** | **A** | **C** | **G** | **T** | **Location** |
| --- | --- | --- | --- | --- | --- | --- | --- | --- |
| **1** | [5413--5443](https://tandem.bu.edu/trf/output/tmpafrwxcqb.2.7.7.80.10.50.500.1.txt.html#5413--5443,15,2.1,15,1) | 30 | 2×15 | 32 | 0 | 12 | 54 | *rps16 (Intron)* |
| **2** | [6002--6026](https://tandem.bu.edu/trf/output/tmpafrwxcqb.2.7.7.80.10.50.500.1.txt.html#6002--6026,13,1.9,13,2) | 26 | 2×13 | 48 | 0 | 36 | 16 | *rps16 trnQ-UUG/ (IGS)* |
| **3** | [6883--6914](https://tandem.bu.edu/trf/output/tmpafrwxcqb.2.7.7.80.10.50.500.1.txt.html#6883--6914,11,3.1,11,3) | 33 | 3×11 | 28 | 9 | 9 | 53 | *trnQ-UUG/psbK (IGS)* |
| **4** | [30676--30718](https://tandem.bu.edu/trf/output/tmpafrwxcqb.2.7.7.80.10.50.500.1.txt.html#30676--30718,16,2.8,16,4) | 48 | 3×16 | 16 | 20 | 0 | 62 | *trnE-UUC/ trnT-GGU (IGS)* |
| **5** | [35751--35782](https://tandem.bu.edu/trf/output/tmpafrwxcqb.2.7.7.80.10.50.500.1.txt.html#35751--35782,16,2.0,16,5) | 32 | 2×16 | 37 | 12 | 12 | 37 | *psbZ/trnG-GCC (IGS)* |
| **6** | [45290--45348](https://tandem.bu.edu/trf/output/tmpafrwxcqb.2.7.7.80.10.50.500.1.txt.html#45290--45348,10,5.8,10,6) | 60 | 6×10 | 28 | 3 | 1 | 66 | *rps4/trnT-UGU (IGS)* |
| **7** | [45593--45648](https://tandem.bu.edu/trf/output/tmpafrwxcqb.2.7.7.80.10.50.500.1.txt.html#45593--45648,28,2.0,28,7) | 56 | 2×28 | 42 | 10 | 7 | 39 | *trnT-UGU /trnL-UAA (IGS)* |
| **8** | [55479--55625](https://tandem.bu.edu/trf/output/tmpafrwxcqb.2.7.7.80.10.50.500.1.txt.html#55479--55625,75,2.0,75,8) | 150 | 2×75 | 32 | 14 | 32 | 19 | *rbcL/accD (IGS)* |
| **9** | [55546--55732](https://tandem.bu.edu/trf/output/tmpafrwxcqb.2.7.7.80.10.50.500.1.txt.html#55546--55732,66,2.8,66,9) | 198 | 3×66 | 28 | 16 | 33 | 21 | *rbcL/accD (IGS)* |
| **10** | [56135--56198](https://tandem.bu.edu/trf/output/tmpafrwxcqb.2.7.7.80.10.50.500.1.txt.html#56135--56198,15,3.9,15,10) | 60 | 4×15 | 25 | 10 | 29 | 34 | *accD (CDS)* |
| **11** | [56555--56748](https://tandem.bu.edu/trf/output/tmpafrwxcqb.2.7.7.80.10.50.500.1.txt.html#56555--56748,96,2.0,96,22) | 192 | 2×96 | 25 | 11 | 31 | 31 | *accD (CDS)* |
| **12** | [57375--57399](https://tandem.bu.edu/trf/output/tmpafrwxcqb.2.7.7.80.10.50.500.1.txt.html#57375--57399,12,2.1,12,26) | 24 | 2×12 | 52 | 8 | 8 | 32 | *accD/psaI (IGS)* |
| **13** | [64265--64296](https://tandem.bu.edu/trf/output/tmpafrwxcqb.2.7.7.80.10.50.500.1.txt.html#64265--64296,16,2.0,16,27) | 32 | 2×16 | 37 | 6 | 15 | 40 | *petL/petG (IGS)* |
| **14** | [64659--64684](https://tandem.bu.edu/trf/output/tmpafrwxcqb.2.7.7.80.10.50.500.1.txt.html#64659--64684,13,2.0,13,28) | 26 | 2×13 | 30 | 0 | 7 | 61 | *trnW-CCA/trnP-UGG (IGS)* |
| **15** | [66205--66286](https://tandem.bu.edu/trf/output/tmpafrwxcqb.2.7.7.80.10.50.500.1.txt.html#66205--66286,21,3.9,21,29) | 84 | 4×21 | 29 | 28 | 13 | 29 | *rps18 (CDS)* |
| **16** | [74595--74634](https://tandem.bu.edu/trf/output/tmpafrwxcqb.2.7.7.80.10.50.500.1.txt.html#74595--74634,19,2.1,19,32) | 38 | 2×19 | 37 | 15 | 0 | 47 | *petD (Intron)* |
| **17** | [77820--77850](https://tandem.bu.edu/trf/output/tmpafrwxcqb.2.7.7.80.10.50.500.1.txt.html#77820--77850,12,2.6,12,33) | 24 | 2×12 | 29 | 12 | 6 | 51 | *infA/rps8 (IGS)* |
| **18** | [81617--81686](https://tandem.bu.edu/trf/output/tmpafrwxcqb.2.7.7.80.10.50.500.1.txt.html#81617--81686,18,4.1,18,34) | 72 | 4×18 | 14 | 45 | 12 | 27 | *rpl22 (CDS)* |
| **19** | [84992--85039](https://tandem.bu.edu/trf/output/tmpafrwxcqb.2.7.7.80.10.50.500.1.txt.html#84992--85039,12,4.0,12,35) | 48 | 4×12 | 41 | 0 | 33 | 25 | *ycf2 (CDS)* |
| **20** | [88987--89060](https://tandem.bu.edu/trf/output/tmpafrwxcqb.2.7.7.80.10.50.500.1.txt.html#88987--89060,18,4.4,18,40) | 72 | 4×18 | 24 | 12 | 35 | 28 | *ycf2 (CDS)* |
| **21** | [96204--96229](https://tandem.bu.edu/trf/output/tmpafrwxcqb.2.7.7.80.10.50.500.1.txt.html#96204--96229,13,2.0,13,41) | 26 | 2×13 | 69 | 7 | 15 | 7 | *rps12/ trnV-GAC (IGS)* |
| **22** | [96683--96716](https://tandem.bu.edu/trf/output/tmpafrwxcqb.2.7.7.80.10.50.500.1.txt.html#96683--96716,17,2.0,17,42) | 34 | 2×17 | 26 | 2 | 23 | 47 | *rps12/ trnV-GAC (IGS)* |
| **23** | [106208--106307](https://tandem.bu.edu/trf/output/tmpafrwxcqb.2.7.7.80.10.50.500.1.txt.html#106208--106307,49,2.2,43,43) | 86 | 2×43 | 55 | 10 | 11 | 24 | *trnN-GUU/ycf1 (IGS)* |
| **24** | [108761--108802](https://tandem.bu.edu/trf/output/tmpafrwxcqb.2.7.7.80.10.50.500.1.txt.html#108761--108802,20,2.0,21,44) | 42 | 2×21 | 54 | 23 | 0 | 21 | *ndhF (CDS)* |
| **25** | [109783--109813](https://tandem.bu.edu/trf/output/tmpafrwxcqb.2.7.7.80.10.50.500.1.txt.html#109783--109813,15,2.1,15,46) | 30 | 2×15 | 32 | 0 | 6 | 61 | *ndhF/ rpl32 (IGS)* |
| **26** | [117879--117924](https://tandem.bu.edu/trf/output/tmpafrwxcqb.2.7.7.80.10.50.500.1.txt.html#117879--117924,23,2.0,23,47) | 46 | 2×23 | 39 | 17 | 13 | 30 | *ndhA (Intron)* |
| **27** | [118122--118158](https://tandem.bu.edu/trf/output/tmpafrwxcqb.2.7.7.80.10.50.500.1.txt.html#118122--118158,18,2.1,18,48) | 36 | 2×18 | 27 | 16 | 13 | 43 | *ndhA (Intron)* |
| **28** | [122721--122784](https://tandem.bu.edu/trf/output/tmpafrwxcqb.2.7.7.80.10.50.500.1.txt.html#122721--122784,33,1.9,33,49) | 66 | 2×33 | 42 | 9 | 14 | 34 | *ycf 1 (CDS)* |
| **29** | [135866--135899](https://tandem.bu.edu/trf/output/tmpafrwxcqb.2.7.7.80.10.50.500.1.txt.html#135866--135899,17,2.0,17,50) | 34 | 2×17 | 47 | 23 | 2 | 26 | *trnV-GAC/rps12 (IGS)* |
| **30** | [136353--136378](https://tandem.bu.edu/trf/output/tmpafrwxcqb.2.7.7.80.10.50.500.1.txt.html#136353--136378,13,2.0,13,51) | 26 | 2×13 | 7 | 15 | 7 | 69 | *trnV-GAC/rps12 (IGS)* |
| **32** | [143522--143595](https://tandem.bu.edu/trf/output/tmpafrwxcqb.2.7.7.80.10.50.500.1.txt.html#143522--143595,18,4.4,18,52) | 72 | 4×18 | 28 | 35 | 12 | 24 | *ycf2 (CDS)* |
| **33** | [147543--147590](https://tandem.bu.edu/trf/output/tmpafrwxcqb.2.7.7.80.10.50.500.1.txt.html#147543--147590,12,4.0,12,56) | 48 | 4×12 | 25 | 33 | 0 | 41 | *ycf2 (CDS)* |

**Table S11a**. Tandem Repeat sequences in the *Plantago atrata*  chloroplast genome.

| **Serial no** | **Indices** | **Repeat**  **Length** | **Size of repeat unit × Copy number** | **A** | **C** | **G** | **T** | **Location** |
| --- | --- | --- | --- | --- | --- | --- | --- | --- |
| **1** | [5712--5741](https://tandem.bu.edu/trf/output/tmp1bel_u2w.2.7.7.80.10.50.500.1.txt.html#5712--5741,15,2.0,15,1) | 30 | 2×15 | 33 | 0 | 13 | 53 | *rps16 (Intron)* |
| **2** | [6421--6454](https://tandem.bu.edu/trf/output/tmp1bel_u2w.2.7.7.80.10.50.500.1.txt.html#6421--6454,15,2.3,15,2) | 30 | 2×15 | 35 | 11 | 0 | 52 | *rps16/trnQ-UUG (IGS)* |
| **3** | [8234--8307](https://tandem.bu.edu/trf/output/tmp1bel_u2w.2.7.7.80.10.50.500.1.txt.html#8234--8307,38,1.9,38,3) | 76 | 2×38 | 44 | 9 | 21 | 24 | *trnS-GCU/trnG-UCC (IGS)* |
| **4** | [12918--12954](https://tandem.bu.edu/trf/output/tmp1bel_u2w.2.7.7.80.10.50.500.1.txt.html#12918--12954,18,2.1,18,5) | 36 | 2×18 | 56 | 2 | 5 | 35 | *atpF/atpH (IGS)* |
| **5** | [29695--29741](https://tandem.bu.edu/trf/output/tmp1bel_u2w.2.7.7.80.10.50.500.1.txt.html#29695--29741,16,2.9,16,6) | 48 | 3×16 | 44 | 10 | 17 | 27 | *psbM/trnD-GUC (IGS)* |
| **6** | [34662--34687](https://tandem.bu.edu/trf/output/tmp1bel_u2w.2.7.7.80.10.50.500.1.txt.html#34662--34687,13,2.0,13,7) | 26 | 2×13 | 46 | 0 | 0 | 53 | *psbC/trnS-UGA (IGS)* |
| **7** | [41255--41290](https://tandem.bu.edu/trf/output/tmp1bel_u2w.2.7.7.80.10.50.500.1.txt.html#41255--41290,18,2.0,18,8) | 36 | 2×18 | 13 | 11 | 8 | 66 | *psaA /ycf3 (IGS)* |
| **8** | [45164--45195](https://tandem.bu.edu/trf/output/tmp1bel_u2w.2.7.7.80.10.50.500.1.txt.html#45164--45195,16,2.0,16,9) | 32 | 2×16 | 62 | 0 | 25 | 12 | *rps4/trnT-UGU (IGS)* |
| **9** | [45232--45272](https://tandem.bu.edu/trf/output/tmp1bel_u2w.2.7.7.80.10.50.500.1.txt.html#45232--45272,21,2.0,21,10) | 42 | 2×21 | 29 | 7 | 4 | 58 | *rps4/trnT-UGU (IGS)* |
| **10** | [55729--56028](https://tandem.bu.edu/trf/output/tmp1bel_u2w.2.7.7.80.10.50.500.1.txt.html#55729--56028,66,4.5,66,11) | 330 | 5×66 | 27 | 17 | 34 | 20 | *rbcL/accD (IGS)* |
| **11** | [56440--56481](https://tandem.bu.edu/trf/output/tmp1bel_u2w.2.7.7.80.10.50.500.1.txt.html#56440--56481,15,2.8,15,13) | 45 | 3×15 | 28 | 7 | 28 | 35 | *accD (CDS)* |
| **12** | [56914--56943](https://tandem.bu.edu/trf/output/tmp1bel_u2w.2.7.7.80.10.50.500.1.txt.html#56914--56943,15,2.0,15,21) | 30 | 2×15 | 26 | 6 | 33 | 33 | *accD (CDS)* |
| **13** | [64585--64616](https://tandem.bu.edu/trf/output/tmp1bel_u2w.2.7.7.80.10.50.500.1.txt.html#64585--64616,16,2.0,16,22) | 32 | 2×16 | 37 | 6 | 15 | 40 | *petL/petG (IGS)* |
| **14** | [66491--66551](https://tandem.bu.edu/trf/output/tmp1bel_u2w.2.7.7.80.10.50.500.1.txt.html#66491--66551,21,2.9,21,23) | 63 | 3×21 | 29 | 27 | 13 | 29 | *rps18 (CDS)* |
| **15** | [72794--72834](https://tandem.bu.edu/trf/output/tmp1bel_u2w.2.7.7.80.10.50.500.1.txt.html#72794--72834,21,1.9,22,24) | 44 | 2×22 | 48 | 4 | 12 | 34 | *petD (Intron)* |
| **16** | [77816--77871](https://tandem.bu.edu/trf/output/tmp1bel_u2w.2.7.7.80.10.50.500.1.txt.html#77816--77871,26,2.2,26,25) | 52 | 2×26 | 37 | 12 | 3 | 46 | *infA/rps8 (IGS)* |
| **17** | [77954--78005](https://tandem.bu.edu/trf/output/tmp1bel_u2w.2.7.7.80.10.50.500.1.txt.html#77954--78005,20,2.7,19,26) | 57 | 3×19 | 40 | 5 | 3 | 50 | *infA/rps8 (IGS)* |
| **18** | [77965--77994](https://tandem.bu.edu/trf/output/tmp1bel_u2w.2.7.7.80.10.50.500.1.txt.html#77965--77994,15,2.0,15,27) | 30 | 2×15 | 43 | 3 | 6 | 46 | *infA/rps8 (IGS)* |
| **19** | [78089--78121](https://tandem.bu.edu/trf/output/tmp1bel_u2w.2.7.7.80.10.50.500.1.txt.html#78089--78121,15,2.2,15,29) | 30 | 2×15 | 36 | 15 | 3 | 45 | *infA/rps8 (IGS)* |
| **20** | [85094--85136](https://tandem.bu.edu/trf/output/tmp1bel_u2w.2.7.7.80.10.50.500.1.txt.html#85094--85136,18,2.4,17,30) | 34 | 2×17 | 58 | 9 | 6 | 25 | *trnM-CAU/ycf2 (IGS)* |
| **21** | [86532--86596](https://tandem.bu.edu/trf/output/tmp1bel_u2w.2.7.7.80.10.50.500.1.txt.html#86532--86596,21,3.1,21,31) | 63 | 3×21 | 24 | 16 | 18 | 40 | *ycf2 (CDS)* |
| **22** | [89152--89225](https://tandem.bu.edu/trf/output/tmp1bel_u2w.2.7.7.80.10.50.500.1.txt.html#89152--89225,18,4.4,18,32) | 72 | 4×18 | 24 | 12 | 35 | 28 | *ycf2 (CDS)* |
| **23** | [96806--96857](https://tandem.bu.edu/trf/output/tmp1bel_u2w.2.7.7.80.10.50.500.1.txt.html#96806--96857,23,2.2,23,33) | 46 | 2×23 | 26 | 1 | 15 | 55 | *rps12/ trnV-GAC (IGS)* |
| **24** | [96839--96872](https://tandem.bu.edu/trf/output/tmp1bel_u2w.2.7.7.80.10.50.500.1.txt.html#96839--96872,17,2.0,17,34) | 34 | 2×17 | 26 | 2 | 23 | 47 | *rps12/ trnV-GAC (IGS)* |
| **25** | [97446--97478](https://tandem.bu.edu/trf/output/tmp1bel_u2w.2.7.7.80.10.50.500.1.txt.html#97446--97478,16,2.1,16,35) | 32 | 2×16 | 30 | 12 | 27 | 30 | *rps12/ trnV-GAC (IGS)* |
| **26** | [108864--108905](https://tandem.bu.edu/trf/output/tmp1bel_u2w.2.7.7.80.10.50.500.1.txt.html#108864--108905,20,2.0,21,36) | 42 | 2×21 | 54 | 23 | 0 | 21 | *ndhF (CDS)* |
| **27** | [120117--120141](https://tandem.bu.edu/trf/output/tmp1bel_u2w.2.7.7.80.10.50.500.1.txt.html#120117--120141,11,2.3,11,38) | 22 | 2×11 | 28 | 0 | 24 | 48 | *rps15/ ycf1 (IGS)* |
| **28** | [120398--120442](https://tandem.bu.edu/trf/output/tmp1bel_u2w.2.7.7.80.10.50.500.1.txt.html#120398--120442,13,3.4,13,39) | 39 | 3×13 | 48 | 0 | 13 | 37 | *rps15/ ycf1 (IGS)* |
| **29** | [121286--121332](https://tandem.bu.edu/trf/output/tmp1bel_u2w.2.7.7.80.10.50.500.1.txt.html#121286--121332,15,3.1,16,40) | 48 | 3×16 | 23 | 10 | 4 | 61 | *ycf 1 (CDS)* |
| **30** | [122479--122542](https://tandem.bu.edu/trf/output/tmp1bel_u2w.2.7.7.80.10.50.500.1.txt.html#122479--122542,33,1.9,33,41) | 66 | 2×33 | 42 | 7 | 14 | 35 | *ycf 1 (CDS)* |
| **31** | [134584--134616](https://tandem.bu.edu/trf/output/tmp1bel_u2w.2.7.7.80.10.50.500.1.txt.html#134584--134616,16,2.1,16,45) | 32 | 2×16 | 30 | 27 | 12 | 30 | *trnV-GAC/rps12 (IGS)* |
| **32** | [135190--135223](https://tandem.bu.edu/trf/output/tmp1bel_u2w.2.7.7.80.10.50.500.1.txt.html#135190--135223,17,2.0,17,46) | 34 | 2×17 | 47 | 23 | 2 | 26 | *trnV-GAC/rps12 (IGS)* |
| **33** | [135205--135256](https://tandem.bu.edu/trf/output/tmp1bel_u2w.2.7.7.80.10.50.500.1.txt.html#135205--135256,23,2.2,23,47) | 46 | 2×23 | 55 | 15 | 1 | 26 | *trnV-GAC/rps12 (IGS)* |
| **34** | [142837--142910](https://tandem.bu.edu/trf/output/tmp1bel_u2w.2.7.7.80.10.50.500.1.txt.html#142837--142910,18,4.4,18,48) | 72 | 4×18 | 28 | 35 | 12 | 24 | *ycf2 (CDS)* |
| **35** | [145466--145530](https://tandem.bu.edu/trf/output/tmp1bel_u2w.2.7.7.80.10.50.500.1.txt.html#145466--145530,21,3.1,21,49) | 42 | 2×21 | 40 | 18 | 16 | 24 | *ycf2 (CDS)* |
| **36** | [146926--146968](https://tandem.bu.edu/trf/output/tmp1bel_u2w.2.7.7.80.10.50.500.1.txt.html#146926--146968,18,2.4,17,50) | 34 | 2×17 | 25 | 6 | 9 | 58 | *ycf2/ trnM-CAU (IGS)* |

**Table S11b**. Tandem Repeat sequences in the *Plantago lanceolata* chloroplast genome.

| **Serial no** | **Indices** | **Repeat**  **Length** | **Size of repeat unit × Copy number** | **A** | **C** | **G** | **T** | **Location** |
| --- | --- | --- | --- | --- | --- | --- | --- | --- |
| **1** | [5417--5448](https://tandem.bu.edu/trf/output/tmp2844t745.2.7.7.80.10.50.500.1.txt.html#5417--5448,15,2.1,16,1) | 32 | 2×16 | 37 | 0 | 6 | 56 | *rps16 (Intron)* |
| **2** | [6893--6924](https://tandem.bu.edu/trf/output/tmp2844t745.2.7.7.80.10.50.500.1.txt.html#6893--6924,11,3.1,11,2) | 33 | 3×11 | 28 | 9 | 9 | 53 | *trnQ-UUG/psbK (IGS)* |
| **3** | [8104--8177](https://tandem.bu.edu/trf/output/tmp2844t745.2.7.7.80.10.50.500.1.txt.html#8104--8177,38,1.9,38,4) | 76 | 2×38 | 45 | 9 | 18 | 25 | *trnS-GCU/trnG-UCC (IGS)* |
| **4** | [8413--8441](https://tandem.bu.edu/trf/output/tmp2844t745.2.7.7.80.10.50.500.1.txt.html#8413--8441,10,3.0,10,6) | 30 | 3×10 | 51 | 0 | 10 | 37 | *trnS-GCU/trnG-UCC (IGS)* |
| **5** | [35713--35744](https://tandem.bu.edu/trf/output/tmp2844t745.2.7.7.80.10.50.500.1.txt.html#35713--35744,16,2.0,16,7) | 32 | 2×16 | 37 | 12 | 12 | 37 | *psbZ/trnG-GCC (IGS)* |
| **6** | [55146--55173](https://tandem.bu.edu/trf/output/tmp2844t745.2.7.7.80.10.50.500.1.txt.html#55146--55173,13,2.2,13,9) | 26 | 2×13 | 7 | 0 | 0 | 92 | *rbcL/accD (IGS)* |
| **7** | [55382--55593](https://tandem.bu.edu/trf/output/tmp2844t745.2.7.7.80.10.50.500.1.txt.html#55382--55593,66,2.9,75,10) | 225 | 3×75 | 32 | 16 | 32 | 19 | *rbcL/accD (IGS)* |
| **8** | [55449--55697](https://tandem.bu.edu/trf/output/tmp2844t745.2.7.7.80.10.50.500.1.txt.html#55449--55697,66,3.8,66,16) | 264 | 4×66 | 28 | 16 | 32 | 21 | *rbcL/accD (IGS)* |
| **9** | [56098--56143](https://tandem.bu.edu/trf/output/tmp2844t745.2.7.7.80.10.50.500.1.txt.html#56098--56143,15,3.1,15,18) | 45 | 3×15 | 26 | 8 | 28 | 36 | *accD (CDS)* |
| **10** | [56299--56394](https://tandem.bu.edu/trf/output/tmp2844t745.2.7.7.80.10.50.500.1.txt.html#56299--56394,48,2.0,48,19) | 96 | 2×48 | 32 | 12 | 34 | 20 | *accD (CDS)* |
| **11** | [56538--56693](https://tandem.bu.edu/trf/output/tmp2844t745.2.7.7.80.10.50.500.1.txt.html#56538--56693,51,3.2,51,28) | 153 | 3×51 | 25 | 9 | 32 | 33 | *accD (CDS)* |
| **12** | [63678--63722](https://tandem.bu.edu/trf/output/tmp2844t745.2.7.7.80.10.50.500.1.txt.html#63678--63722,13,3.2,14,29) | 42 | 3×14 | 44 | 2 | 2 | 51 | *psbE/petL (IGS)* |
| **13** | [63680--63730](https://tandem.bu.edu/trf/output/tmp2844t745.2.7.7.80.10.50.500.1.txt.html#63680--63730,15,3.5,15,30) | 60 | 4×15 | 41 | 1 | 3 | 52 | *psbE/petL (IGS)* |
| **14** | [64204--64235](https://tandem.bu.edu/trf/output/tmp2844t745.2.7.7.80.10.50.500.1.txt.html#64204--64235,16,2.0,16,31) | 32 | 2×16 | 37 | 6 | 15 | 40 | *petL/petG (IGS)* |
| **15** | [66145--66205](https://tandem.bu.edu/trf/output/tmp2844t745.2.7.7.80.10.50.500.1.txt.html#66145--66205,21,2.9,21,32) | 63 | 3×21 | 29 | 27 | 13 | 29 | *rps18 (CDS)* |
| **16** | [77589--77616](https://tandem.bu.edu/trf/output/tmp2844t745.2.7.7.80.10.50.500.1.txt.html#77589--77616,14,2.0,14,33) | 28 | 2×14 | 35 | 21 | 7 | 35 | *infA/rps8 (IGS)* |
| **17** | [81519--81588](https://tandem.bu.edu/trf/output/tmp2844t745.2.7.7.80.10.50.500.1.txt.html#81519--81588,18,4.1,18,34) | 72 | 4×18 | 14 | 45 | 12 | 27 | *rpl22 (CDS)* |
| **18** | [84893--84928](https://tandem.bu.edu/trf/output/tmp2844t745.2.7.7.80.10.50.500.1.txt.html#84893--84928,12,3.0,12,36) | 36 | 3×12 | 41 | 0 | 33 | 25 | *ycf2 (CDS)* |
| **19** | [88882--88955](https://tandem.bu.edu/trf/output/tmp2844t745.2.7.7.80.10.50.500.1.txt.html#88882--88955,18,4.4,18,40) | 72 | 4×18 | 24 | 12 | 35 | 28 | *ycf2 (CDS)* |
| **20** | [96099--96124](https://tandem.bu.edu/trf/output/tmp2844t745.2.7.7.80.10.50.500.1.txt.html#96099--96124,13,2.0,13,41) | 26 | 2×13 | 69 | 7 | 15 | 7 | *rps12/ trnV-GAC (IGS)* |
| **21** | [96578--96611](https://tandem.bu.edu/trf/output/tmp2844t745.2.7.7.80.10.50.500.1.txt.html#96578--96611,17,2.0,17,42) | 34 | 2×17 | 26 | 2 | 23 | 47 | *rps12/ trnV-GAC (IGS)* |
| **22** | [106018--106147](https://tandem.bu.edu/trf/output/tmp2844t745.2.7.7.80.10.50.500.1.txt.html#106018--106147,42,2.6,53,43) | 159 | 3×53 | 61 | 9 | 10 | 18 | *trnN-GUU/ycf1 (IGS)* |
| **23** | [106047--106138](https://tandem.bu.edu/trf/output/tmp2844t745.2.7.7.80.10.50.500.1.txt.html#106047--106138,42,2.2,42,44) | 84 | 2×42 | 57 | 9 | 10 | 21 | *trnN-GUU/ycf1 (IGS)* |
| **24** | [108592--108633](https://tandem.bu.edu/trf/output/tmp2844t745.2.7.7.80.10.50.500.1.txt.html#108592--108633,20,2.0,21,45) | 42 | 2×21 | 54 | 23 | 0 | 21 | *ndhF (CDS)* |
| **25** | [109613--109643](https://tandem.bu.edu/trf/output/tmp2844t745.2.7.7.80.10.50.500.1.txt.html#109613--109643,15,2.1,15,47) | 30 | 2×15 | 32 | 0 | 6 | 61 | *ndhF/ rpl32 (IGS)* |
| **26** | [118483--118507](https://tandem.bu.edu/trf/output/tmp2844t745.2.7.7.80.10.50.500.1.txt.html#118483--118507,12,2.1,12,48) | 24 | 2×12 | 44 | 16 | 8 | 32 | *ndhA (Exon)* |
| **27** | [121256--121296](https://tandem.bu.edu/trf/output/tmp2844t745.2.7.7.80.10.50.500.1.txt.html#121256--121296,21,2.0,21,50) | 42 | 2×21 | 14 | 14 | 9 | 60 | *ycf 1 (CDS)* |
| **28** | [121349--121380](https://tandem.bu.edu/trf/output/tmp2844t745.2.7.7.80.10.50.500.1.txt.html#121349--121380,16,2.0,16,52) | 32 | 2×16 | 0 | 6 | 6 | 87 | *ycf 1 (CDS)* |
| **29** | [126108--126199](https://tandem.bu.edu/trf/output/tmp2844t745.2.7.7.80.10.50.500.1.txt.html#126108--126199,42,2.2,42,54) | 84 | 2×42 | 21 | 10 | 9 | 57 | *ycf1/ trnN-GUU (IGS)* |
| **30** | [135635--135668](https://tandem.bu.edu/trf/output/tmp2844t745.2.7.7.80.10.50.500.1.txt.html#135635--135668,17,2.0,17,55) | 34 | 2×17 | 47 | 23 | 2 | 26 | *trnV-GAC/rps12 (IGS)* |
| **31** | [136122--136147](https://tandem.bu.edu/trf/output/tmp2844t745.2.7.7.80.10.50.500.1.txt.html#136122--136147,13,2.0,13,56) | 26 | 2×13 | 7 | 15 | 7 | 69 | *trnV-GAC/rps12 (IGS)* |
| **32** | [143291--143364](https://tandem.bu.edu/trf/output/tmp2844t745.2.7.7.80.10.50.500.1.txt.html#143291--143364,18,4.4,18,57) | 72 | 4×18 | 28 | 35 | 12 | 24 | *ycf2 (CDS)* |
| **33** | [145933--146014](https://tandem.bu.edu/trf/output/tmp2844t745.2.7.7.80.10.50.500.1.txt.html#145933--146014,21,3.9,21,59) | 84 | 4×21 | 39 | 18 | 19 | 23 | *ycf2 (CDS)* |
| **34** | [147318--147353](https://tandem.bu.edu/trf/output/tmp2844t745.2.7.7.80.10.50.500.1.txt.html#147318--147353,12,3.0,12,63) | 36 | 3×12 | 25 | 33 | 0 | 41 | *ycf2 (CDS)* |

**Table S11c**. Tandem Repeat sequences in the *Plantago maritima* chloroplast genome.

| **Serial no** | **Indices** | **Repeat**  **Length** | **Size of repeat unit × Copy number** | **A** | **C** | **G** | **T** | **Location** |
| --- | --- | --- | --- | --- | --- | --- | --- | --- |
| **1** | [3709--3733](https://tandem.bu.edu/trf/output/tmpwy766d77.2.7.7.80.10.50.500.1.txt.html) | 24.7 | 13×1.9 | 32 | 36 | 0 | 32 | *trnK-UUU (Intron)* |
| **2** | [6271--6313](https://tandem.bu.edu/trf/output/tmpwy766d77.2.7.7.80.10.50.500.1.txt.html) | 43.7 | 23×1.9 | 30 | 4 | 9 | 55 | *rps16/trnQ-UUG (IGS)* |
| **3** | [6723--6805](https://tandem.bu.edu/trf/output/tmpwy766d77.2.7.7.80.10.50.500.1.txt.html) | 83.7 | 27×3.1 | 14 | 44 | 14 | 26 | *trnQ-UUG (Gene)* |
| **4** | [8107--8179](https://tandem.bu.edu/trf/output/tmpwy766d77.2.7.7.80.10.50.500.1.txt.html) | 72.2 | 38×1.9 | 45 | 9 | 20 | 24 | *trnS-GCU/trnG-UCC (IGS)* |
| **5** | [9487--9518](https://tandem.bu.edu/trf/output/tmpwy766d77.2.7.7.80.10.50.500.1.txt.html) | 31.5 | 15×2.1 | 43 | 3 | 12 | 40 | *trnG-GCC/trnQ-UUG (IGS)* |
| **6** | [12587--12637](https://tandem.bu.edu/trf/output/tmpwy766d77.2.7.7.80.10.50.500.1.txt.html) | 52.8 | 22×2.4 | 43 | 11 | 21 | 23 | *atpF (Intron)* |
| **7** | [20814--20838](https://tandem.bu.edu/trf/output/tmpwy766d77.2.7.7.80.10.50.500.1.txt.html) | 25.2 | 12×2.1 | 28 | 16 | 16 | 40 | *rpoC2/rpoC1 (IGS)* |
| **8** | [29067--29106](https://tandem.bu.edu/trf/output/tmpwy766d77.2.7.7.80.10.50.500.1.txt.html) | 39.9 | 19×2.1 | 45 | 15 | 5 | 35 | *petN/psbM (IGS)* |
| **9** | [29979--30010](https://tandem.bu.edu/trf/output/tmpwy766d77.2.7.7.80.10.50.500.1.txt.html) | 32 | 16×2 | 43 | 12 | 18 | 25 | *PsbM/* *trnD-GUC (IGS)* |
| **10** | [46060--46124](https://tandem.bu.edu/trf/output/tmpwy766d77.2.7.7.80.10.50.500.1.txt.html) | 67.2 | 32×2.1 | 23 | 12 | 9 | 55 | *trnT-UGU/trnL-UAA (IGS)* |
| **11** | [56678--56873](https://tandem.bu.edu/trf/output/tmpwy766d77.2.7.7.80.10.50.500.1.txt.html) | 199.2 | 83×2.4 | 35 | 7 | 18 | 38 | *rbcL/accD (IGS)* |
| **12** | [56841--56929](https://tandem.bu.edu/trf/output/tmpwy766d77.2.7.7.80.10.50.500.1.txt.html) | 92 | 46×2 | 35 | 6 | 16 | 40 | *rbcL/accD (IGS)* |
| **13** | [57137--57219](https://tandem.bu.edu/trf/output/tmpwy766d77.2.7.7.80.10.50.500.1.txt.html) | 84 | 24×3.5 | 33 | 14 | 26 | 25 | *rbcL/accD (IGS)* |
| **14** | [57238--57273](https://tandem.bu.edu/trf/output/tmpwy766d77.2.7.7.80.10.50.500.1.txt.html) | 37.8 | 18×2.1 | 52 | 5 | 25 | 16 | *rbcL/accD (IGS)* |
| **15** | [57330--57461](https://tandem.bu.edu/trf/output/tmpwy766d77.2.7.7.80.10.50.500.1.txt.html) | 129.6 | 54×2.4 | 37 | 12 | 29 | 20 | *rbcL/accD (IGS)* |
| **16** | [57503--57563](https://tandem.bu.edu/trf/output/tmpwy766d77.2.7.7.80.10.50.500.1.txt.html) | 60.9 | 21×2.9 | 32 | 18 | 32 | 16 | *rbcL/accD (IGS)* |
| **17** | [57522--57563](https://tandem.bu.edu/trf/output/tmpwy766d77.2.7.7.80.10.50.500.1.txt.html) | 42 | 21×2 | 33 | 19 | 33 | 14 | *rbcL/accD (IGS)* |
| **18** | [58272--58385](https://tandem.bu.edu/trf/output/tmpwy766d77.2.7.7.80.10.50.500.1.txt.html) | 114 | 57×2 | 31 | 16 | 21 | 30 | *accD (CDS)* |
| **19** | [58363--58400](https://tandem.bu.edu/trf/output/tmpwy766d77.2.7.7.80.10.50.500.1.txt.html) | 37.5 | 15×2.5 | 42 | 2 | 28 | 26 | *accD (CDS)* |
| **20** | [58997--59022](https://tandem.bu.edu/trf/output/tmpwy766d77.2.7.7.80.10.50.500.1.txt.html) | 26 | 13×2 | 30 | 7 | 15 | 46 | *accD psaI (IGS)* |
| **21** | [63106--63134](https://tandem.bu.edu/trf/output/tmpwy766d77.2.7.7.80.10.50.500.1.txt.html) | 29.4 | 14×2.1 | 58 | 0 | 20 | 20 | *petA/psbJ (IGS)* |
| **22** | [65176--65211](https://tandem.bu.edu/trf/output/tmpwy766d77.2.7.7.80.10.50.500.1.txt.html) | 35.2 | 16×2.2 | 30 | 5 | 11 | 52 | *petL/petG (IGS)* |
| **23** | [65938--65964](https://tandem.bu.edu/trf/output/tmpwy766d77.2.7.7.80.10.50.500.1.txt.html) | 27 | 3×9 | 66 | 0 | 0 | 33 | *trnP-UGG/psaJ (IGS)* |
| **24** | [66350--66389](https://tandem.bu.edu/trf/output/tmpwy766d77.2.7.7.80.10.50.500.1.txt.html) | 40.8 | 17×2.4 | 5 | 20 | 0 | 75 | *psaJ/* *rpl33 (IGS)* |
| **25** | [67153--67192](https://tandem.bu.edu/trf/output/tmpwy766d77.2.7.7.80.10.50.500.1.txt.html) | 39.9 | 21×1.9 | 30 | 27 | 12 | 30 | *rps18 (CDS)* |
| **26** | [75293--75347](https://tandem.bu.edu/trf/output/tmpwy766d77.2.7.7.80.10.50.500.1.txt.html) | 54 | 27×2 | 29 | 3 | 14 | 52 | *petD/rpoA (IGS)* |
| **27** | [77585--77723](https://tandem.bu.edu/trf/output/tmpwy766d77.2.7.7.80.10.50.500.1.txt.html) | 136.5 | 21×6.5 | 12 | 23 | 6 | 58 | *infA/rps8 (IGS)* |
| **28** | [83179--83224](https://tandem.bu.edu/trf/output/tmpwy766d77.2.7.7.80.10.50.500.1.txt.html) | 46 | 23×2 | 54 | 8 | 15 | 21 | *rpl23/ trnI-CAU (IGS)* |
| **29** | [83258--83370](https://tandem.bu.edu/trf/output/tmpwy766d77.2.7.7.80.10.50.500.1.txt.html) | 112 | 56×2 | 38 | 12 | 23 | 26 | *rpl23/ trnI-CAU (IGS)* |
| **30** | [85052--85154](https://tandem.bu.edu/trf/output/tmpwy766d77.2.7.7.80.10.50.500.1.txt.html) | 102 | 51×2 | 23 | 17 | 23 | 35 | *ycf2 (CDS)* |
| **31** | [86304--86330](https://tandem.bu.edu/trf/output/tmpwy766d77.2.7.7.80.10.50.500.1.txt.html) | 26.4 | 12×2.2 | 22 | 7 | 14 | 55 | *ycf2 (CDS)* |
| **32** | [87578--87637](https://tandem.bu.edu/trf/output/tmpwy766d77.2.7.7.80.10.50.500.1.txt.html) | 66.6 | 18×3.7 | 25 | 15 | 33 | 26 | *ycf2 (CDS)* |
| **33** | [89900--90017](https://tandem.bu.edu/trf/output/tmpwy766d77.2.7.7.80.10.50.500.1.txt.html) | 118 | 59×2 | 33 | 21 | 16 | 27 | *trnL-CAA/ycf1 (IGS)* |
| **34** | [94153--94186](https://tandem.bu.edu/trf/output/tmpwy766d77.2.7.7.80.10.50.500.1.txt.html) | 34.5 | 15×2.3 | 61 | 0 | 11 | 26 | *ycf1 (CDS)* |
| **35** | [97850--97910](https://tandem.bu.edu/trf/output/tmpwy766d77.2.7.7.80.10.50.500.1.txt.html) | 62 | 31×2 | 27 | 9 | 24 | 37 | *rrn5/rrn4.5 (IGS)* |
| **36** | [117340--117381](https://tandem.bu.edu/trf/output/tmpwy766d77.2.7.7.80.10.50.500.1.txt.html) | 40 | 20×2 | 54 | 21 | 0 | 23 | *ndhF (CDS)* |
| **37** | [119523--119815](https://tandem.bu.edu/trf/output/tmpwy766d77.2.7.7.80.10.50.500.1.txt.html) | 294 | 147×2 | 30 | 21 | 23 | 25 | *rpl32/trnL-UAG (IGS)* |
| **38** | [142056--142116](https://tandem.bu.edu/trf/output/tmpwy766d77.2.7.7.80.10.50.500.1.txt.html) | 62 | 31×2 | 37 | 24 | 9 | 27 | *rrn4.5/ rrn5 (IGS)* |
| **39** | [145780--145813](https://tandem.bu.edu/trf/output/tmpwy766d77.2.7.7.80.10.50.500.1.txt.html) | 34.5 | 15×2.3 | 26 | 11 | 0 | 61 | *ycf1 (CDS)* |
| **40** | [149949--150066](https://tandem.bu.edu/trf/output/tmpwy766d77.2.7.7.80.10.50.500.1.txt.html) | 118 | 59×2 | 27 | 16 | 21 | 33 | *ycf1/ trnL-CAA (IGS)* |
| **41** | [152333--152388](https://tandem.bu.edu/trf/output/tmpwy766d77.2.7.7.80.10.50.500.1.txt.html) | 61.2 | 18×3.4 | 26 | 33 | 16 | 23 | *ycf2 (CDS)* |
| **42** | [153636--153662](https://tandem.bu.edu/trf/output/tmpwy766d77.2.7.7.80.10.50.500.1.txt.html) | 26.4 | 12×2.2 | 55 | 14 | 7 | 22 | *ycf2 (CDS)* |
| **43** | [154812--154914](https://tandem.bu.edu/trf/output/tmpwy766d77.2.7.7.80.10.50.500.1.txt.html) | 102 | 51×2 | 35 | 23 | 17 | 23 | *ycf2 (CDS)* |
| **44** | [156596--156708](https://tandem.bu.edu/trf/output/tmpwy766d77.2.7.7.80.10.50.500.1.txt.html) | 112 | 56×2 | 26 | 23 | 12 | 38 | *trnI-CAU/ rpl23 (IGS)* |
| **45** | [156742--156787](https://tandem.bu.edu/trf/output/tmpwy766d77.2.7.7.80.10.50.500.1.txt.html) | 46 | 23×2 | 21 | 15 | 8 | 54 | *trnI-CAU/ rpl23 (IGS)* |
