# Supplementary material for: Comparative Plastomics of Plantains (Plantago, Plantaginaceae) as a Tool for the Development of Species-Specific DNA Barcodes
Source: Plants (Basel). 2024 Sep 25;13(19):2691. doi: 10.3390/plants13192691 (PMC11478842; doi:10.3390/plants13192691)
Supplement: Supplementary file 1 [file plants-13-02691-s001.zip › plants-3143619-supplementary/Table S2. base composition_01.docx]

**Table S2 -** Base composition in the *Plantago argentea* plastid genome

|  | **A%** | **C %** | **G%** | **T %** | **Length (bp)** |
| --- | --- | --- | --- | --- | --- |
| **Total** | 30.4 | 19.4 | 18.9 | 31.2 | 150,092 |
| **LSC** | 31 | 18.6 | 17.9 | 32.5 | 82,489 |
| **SSC** | 34 | 16.8 | 15.7 | 33.5 | 18,464 |
| **IR** | 28.4 | 20.9 | 22.6 | 28.1 | 24,591 |
| **tRNA** | 24 | 26.5 | 26.3 | 23.2 | 2893 |
| **rRNA** | 22.4 | 27.6 | 27.6 | 22.4 | 9058 |
| **Protein Coding genes** | 30.3 | 19.6 | 18.9 | 31.2 | 77,625 |
| **1st Position** | 30.2 | 19.1 | 19.1 | 31.5 | 50,031 |
| **2nd position** | 30.2 | 19.6 | 19.1 | 31.1 | 50,031 |
| **3rd position** | 30.9 | 19.5 | 18.5 | 31.1 | 50,030 |

**Table S2a -** Base composition in the *Plantago atrata* plastid genome

|  | **A%** | **C%** | **G%** | **T%** | **Length (bp)** |
| --- | --- | --- | --- | --- | --- |
| **Total** | 30.5 | 19.4 | 18.9 | 31.2 | 149,381 |
| **LSC** | 31.1 | 18.5 | 17.9 | 32.5 | 82,680 |
| **SSC** | 34 | 16.8 | 15.5 | 33.7 | 17,671 |
| **IR** | 28.4 | 20.9 | 22.7 | 28.1 | 24,515 |
| **tRNA** | 23.9 | 26.5 | 26.3 | 23.3 | 2883 |
| **rRNA** | 22.4 | 27.6 | 27.6 | 22.4 | 9058 |
| **Protein Coding genes** | 30.2 | 19.6 | 18.9 | 31.3 | 77,109 |
| **1st Position** | 30.4 | 19.4 | 18.8 | 31.4 | 49,794 |
| **2nd position** | 30.8 | 19 | 19 | 31.2 | 49,794 |
| **3rd position** | 31.2 | 19.8 | 18.9 | 31.1 | 49,793 |

**Table S2b -** Base composition in the *Plantago lanceolata* plastid genome

|  | **A%** | **C%** | **G%** | **T%** | **Length (bp)** |
| --- | --- | --- | --- | --- | --- |
| **Total** | 30.5 | 19.4 | 18.9 | 31.2 | 149,832 |
| **LSC** | 31 | 18.6 | 17.9 | 32.4 | 82,413 |
| **SSC** | 34 | 16.8 | 15.8 | 33.4 | 18,421 |
| **IR** | 28.4 | 20.9 | 22.6 | 28.0 | 24,499 |
| **tRNA** | 23.9 | 26.5 | 26.4 | 23.2 | 2891 |
| **rRNA** | 22.4 | 27.6 | 27.6 | 22.4 | 9058 |
| **Protein Coding genes** | 30.3 | 19.6 | 18.9 | 31.2 | 77,586 |
| **1st Position** | 29.6 | 20.2 | 19.1 | 31.2 | 49,944 |
| **2nd position** | 31.3 | 19.0 | 18.3 | 31.4 | 49,944 |
| **3rd position** | 30.6 | 19.1 | 19.4 | 31.0 | 49,944 |

**Table S2c -** Base composition in the *Plantago maritima* plastid genome

|  | **A%** | **C%** | **G%** | **T%** | **Length (bp)** |
| --- | --- | --- | --- | --- | --- |
| **Total** | 30.5 | 19.6 | 19.1 | 30.9 | 149,832 |
| **LSC** | 30.8 | 18.9 | 18.1 | 32.2 | 81,909 |
| **SSC** | 36.8 | 16.8 | 16 | 30.4 | 8,665 |
| **IR** | 29.2 | 20.4 | 20.9 | 29.4 | 33,741 |
| **tRNA** | 23.6 | 27.4 | 26.4 | 22.6 | 2,996 |
| **rRNA** | 22.4 | 27.6 | 27.6 | 22.4 | 9,058 |
| **Protein Coding genes** | 30.4 | 19.7 | 19.1 | 30.8 | 85,266 |
| **1st Position** | 30.7 | 19.7 | 19.1 | 30.6 | 52,686 |
| **2nd position** | 30.2 | 19.2 | 19.1 | 31.1 | 52,685 |
| **3rd position** | 30.6 | 19.4 | 19.1 | 31.0 | 52,685 |
