# Supplementary material for: Comparative Plastomics of Plantains (Plantago, Plantaginaceae) as a Tool for the Development of Species-Specific DNA Barcodes
Source: Plants (Basel). 2024 Sep 25;13(19):2691. doi: 10.3390/plants13192691 (PMC11478842; doi:10.3390/plants13192691)
Supplement: Supplementary file 1 [file plants-13-02691-s001.zip › plants-3143619-supplementary/Table S4 SNPs and Indels comparision.docx]

| **Types** | ***Plantago atrata*** | ***Plantago lanceolata*** | ***Plantago maritima*** |
| --- | --- | --- | --- |
| **C/T** | 114 | 25 | 3 |
| **A/G** | 135 | 34 | 8 |
| **A/T** | 62 | 8 | 2 |
| **A/C** | 61 | 17 | 7 |
| **G/T** | 67 | 17 | 6 |
| **C/G** | 10 | 1 | 2 |
| **Total** | 449 | 102 | 28 |
| **Transition** | 249 | 59 | 11 |
| **Transversion** | 200 | 43 | 17 |
| **Ts/Tv** | 1.245 | 1.372093 | 0.647059 |

**Table S4.** *Plantago lanceolata* (MW877582.1), *Plantago atrata* (MW877580.1) and *Plantago maritima* (KR297244.1) SNPs comparison with our genomes

**Table S4.** *Plantago lanceolata* (MW877582.1), *Plantago atrata* (MW877580.1) and *Plantago maritima* (KR297244.1) indels comparison with our genomes

|  | **InDel sites** | **InDels average length** |
| --- | --- | --- |
| ***Plantago atrata*** | 377 | 4.654 |
| ***Plantago lanceolata*** | 558 | 6.341 |
| ***Plantago maritima*** | 586 | 32.556 |
