# Supplementary figures and images for: Comparative Plastomics of Plantains (Plantago, Plantaginaceae) as a Tool for the Development of Species-Specific DNA Barcodes
Source: Plants (Basel). 2024 Sep 25;13(19):2691. doi: 10.3390/plants13192691 (PMC11478842; doi:10.3390/plants13192691)

Tree scale: 0.1

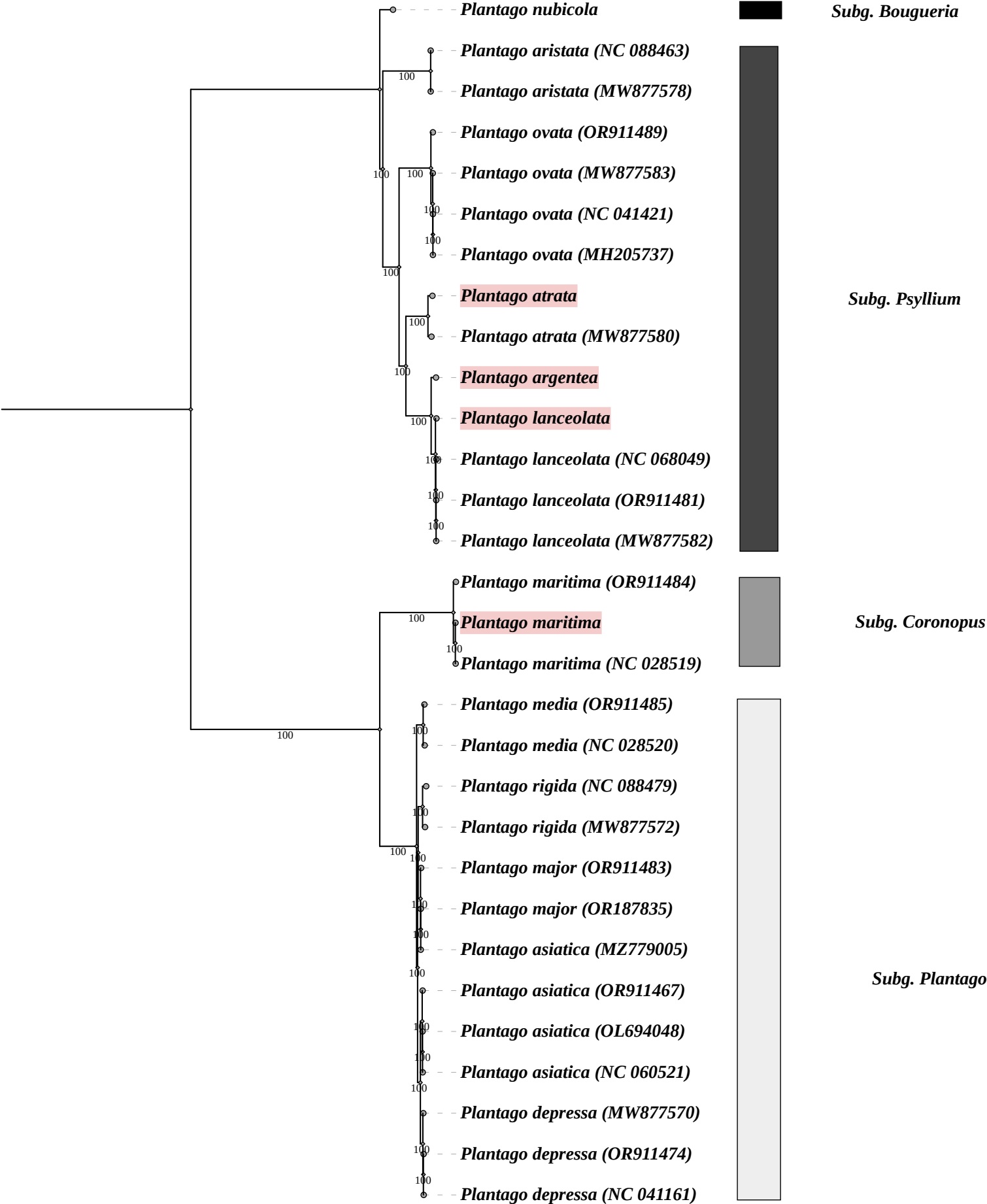

Supplement: Supplementary file 1 [file plants-13-02691-s001.zip › plants-3143619-supplementary/Fig_S1_Multiple_Accession_phylogney.pdf]

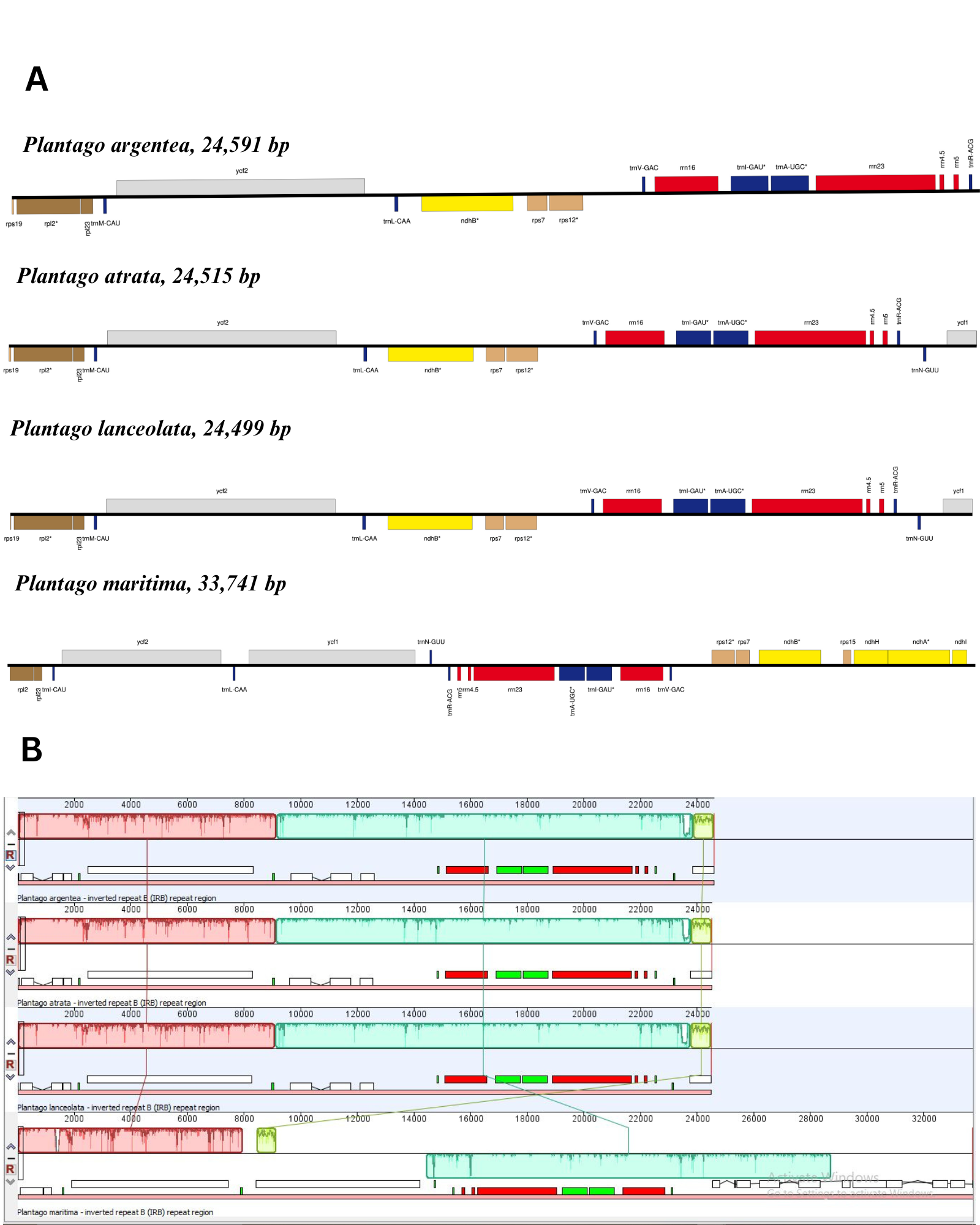

Supplement: Supplementary file 1 [file plants-13-02691-s001.zip › plants-3143619-supplementary/Fig_S2_IR Inversion.png]

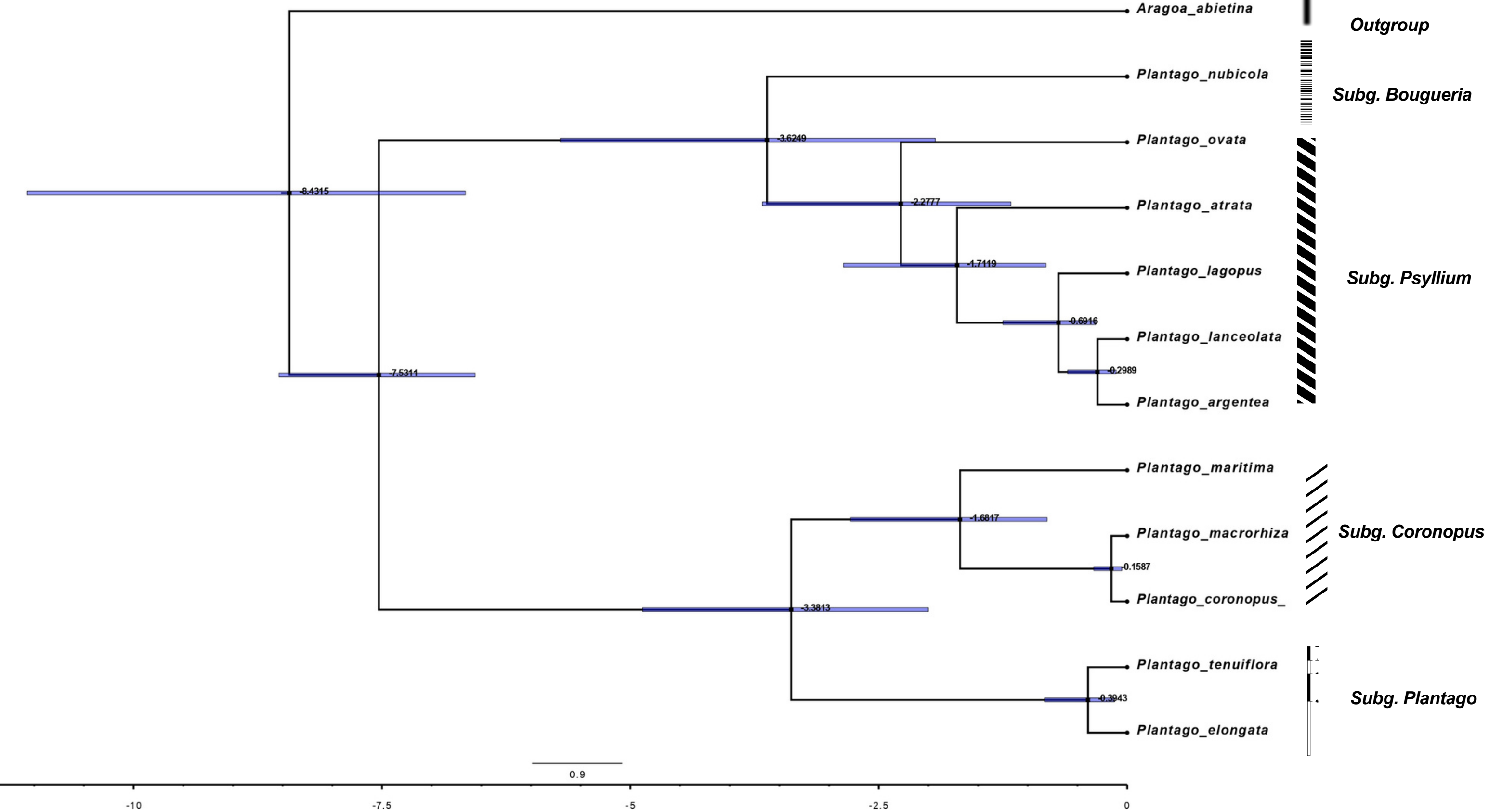

Supplement: Supplementary file 1 [file plants-13-02691-s001.zip › plants-3143619-supplementary/Fig_S3_Plantago FDTT.pdf]
